# Differential connectivity of splicing activators and repressors to the human spliceosome

**DOI:** 10.1186/s13059-015-0682-5

**Published:** 2015-06-06

**Authors:** Martin Akerman, Oliver I. Fregoso, Shipra Das, Cristian Ruse, Mads A. Jensen, Darryl J. Pappin, Michael Q. Zhang, Adrian R. Krainer

**Affiliations:** Cold Spring Harbor Laboratory, Cold Spring Harbor, NY USA; Present address: Envisagenics, Inc, 315 Main St., 2nd floor, Huntington, NY 11743 USA; Watson School of Biological Sciences, Cold Spring Harbor, NY 11724 USA; Department of Molecular and Cell Biology, Center for Systems Biology, The University of Texas at Dallas, Richardson, TX 75080 USA; Bioinformatics Division, TNLIST, Tsinghua University, Beijing, 100084 China; Present address: Fred Hutchinson Cancer Research Center, Division of Human Biology, 1100 Fairview Ave N, Seattle, WA 98109 USA; Present address: New England Biolabs, 240 County Road, Ipswich, MA 01938 UK; Present address: Santaris Pharma A/S, Horsholm, Denmark

## Abstract

**Background:**

During spliceosome assembly, protein-protein interactions (PPI) are sequentially formed and disrupted to accommodate the spatial requirements of pre-mRNA substrate recognition and catalysis. Splicing activators and repressors, such as SR proteins and hnRNPs, modulate spliceosome assembly and regulate alternative splicing. However, it remains unclear how they differentially interact with the core spliceosome to perform their functions.

**Results:**

Here, we investigate the protein connectivity of SR and hnRNP proteins to the core spliceosome using probabilistic network reconstruction based on the integration of interactome and gene expression data. We validate our model by immunoprecipitation and mass spectrometry of the prototypical splicing factors SRSF1 and hnRNPA1. Network analysis reveals that a factor’s properties as an activator or repressor can be predicted from its overall connectivity to the rest of the spliceosome. In addition, we discover and experimentally validate PPIs between the oncoprotein SRSF1 and members of the anti-tumor drug target SF3 complex. Our findings suggest that activators promote the formation of PPIs between spliceosomal sub-complexes, whereas repressors mostly operate through protein-RNA interactions.

**Conclusions:**

This study demonstrates that combining *in-silico* modeling with biochemistry can significantly advance the understanding of structure and function relationships in the human spliceosome.

**Electronic supplementary material:**

The online version of this article (doi:10.1186/s13059-015-0682-5) contains supplementary material, which is available to authorized users.

## Background

The major spliceosome is a biological machine that excises >99 % of human introns. It is composed of approximately 150–300 proteins [1–3], depending on the stage of the splicing reaction and the affinity of proteins for their pre-mRNA substrates [2]. A subset of proteins associate with small nuclear RNAs (snRNAs) to form five small nuclear ribonucleoprotein complexes (snRNPs): U1, U2, U4, U5, and U6. The snRNPs, together with other proteins, constitute the catalytic core of the spliceosome [2, 3]. The spliceosome forms step-wise on the pre-mRNA [2], through sequential rearrangements in which various protein and RNP complexes form and disassemble distinct protein-protein interactions (PPIs), in addition to RNA-RNA and RNA-protein interactions. These transformations, some of which require ATP hydrolysis, are the driving force of splicing catalysis [2, 3].

The structural plasticity of the spliceosome makes it susceptible to regulation, allowing for the skipping or inclusion of alternative exons or exon segments [2], known as alternative splicing. More than 90 % of human primary transcripts undergo alternative splicing [4, 5]. Splicing efficiency and alternative splicing regulation are controlled by *trans*-acting splicing factors, which bind to *cis*-acting elements on the pre-mRNA to either activate or repress the selection of particular splice sites [6].

SR proteins [7] and hnRNPs [8] are two important families of splicing factors. The SR proteins SRSF1-7 typically activate exon inclusion through sequence-specific binding to exonic enhancers [7]. SRSF9-11 share sequence and structure similarity with the rest of the SR family, but they uncharacteristically act as repressors [7]. The hnRNPs are also diverse: a recent study [9] addressing the sequence specificity and splicing activity of five hnRNPs using high-throughput techniques, concluded that hnRNPF, H1, M, and U are primarily activators, whereas hnRNPA1 and A2B1 are primarily repressors.

The regulation of alternative splicing by activators and repressors has been studied by a variety of methods, revealing RNA-binding patterns, cooperative effects, and regulatory targets of particular splicing factors. Although the functions of these factors can be studied in isolation, activators and repressors must work coordinately with the core spliceosome machinery responsible for constitutive and alternative splicing [9–14].

To understand the contextual differences shaping the behavior of activators and repressors, we assembled and studied the PPI networks of all SR proteins and hnRNPs. We conducted a top-down study in three stages: first, we predicted PPIs in the human spliceosome through a probabilistic model that integrates annotated PPIs with gene-expression microarray profiles; second, we implemented the resulting interactome network to investigate the connectivity of SR proteins and hnRNPs to the rest of the spliceosome; and third, we validated the structure of the network by performing immunoprecipitation and mass spectrometry (IP-MS) of two prototypical splicing factors: the activator SRSF1 and the repressor hnRNPA1.

By regarding spliceosomal PPIs as probabilistic (rather than deterministic) events, our model uncovered novel information about the involvement of SR proteins and hnRNPs in splicing regulation. We found that a splicing factor’s property as an activator or repressor can be predicted from its overall connectivity to the spliceosome. Whereas activators (from either the SR or hnRNP families) form several PPIs showing prominent centrality in the spliceosome, repressors are peripheral, and therefore loosely connected to other spliceosomal proteins. We confirmed these observations through IP-MS, and demonstrated that many hnRNPA1 interactions are RNA-dependent, whereas SRSF1 does not require RNA to remain bound to spliceosomal proteins. We discovered that SRSF1 forms multiple PPIs with the early-acting U2-snRNP-specific SF3 complex, which we confirmed by *in-vitro* pull-down experiments. Finally, by combining our data with previously reported co-regulatory interactions, we demonstrate that hnRNPs are distributed in at least two highly interconnected clusters forming regulatory collaborations, consistent with the large cooperativity and functional interchangeability among proteins of this family.

## Results

### A probabilistic model of the human spliceosome

**Fig. 1:**
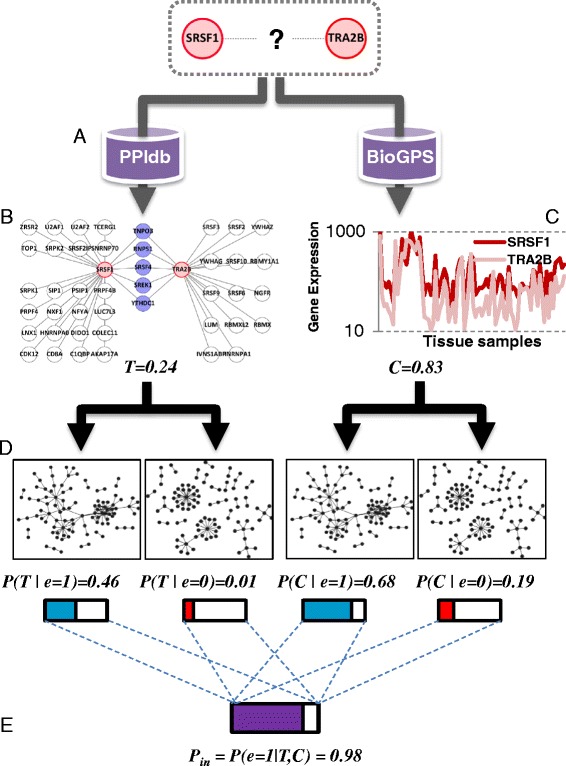
Workflow of the Bayesian probability model to predict protein-protein interactions. Example of how the probability of direct interaction (P_in_) between SRSF1 and TRA2B was calculated. **a** We first extracted all known PPIs formed by SRSF1 or TRA2B from a PPI database. **b** We used the number of shared PPIs between both proteins (blue nodes) and exclusive PPIs (white nodes) to calculate the Transitivity (T). **c** We then extracted their co-expression profile from the BioGPS microarray database and computed the Pearson correlation coefficient (C). **d** By transforming the calculated values of T and C through conditional-probability models, we estimated the probability that both T and C may occur in a true PPI network (e = 1, left network) and a false (that is, shuffled) interactome (e = 0, right network). **e** Finally, the probability P_in_ was calculated using the Bayes rule, as the posterior probability that SRSF1 and TRA2B directly bind each other, given T and C as evidence

The amount of high-quality yeast two-hybrid (Y2H) data has grown remarkably in the last two decades [15], as has the number of analytical methods to interpret PPI networks. Probabilistic modeling is an increasingly popular approach to interrogate PPI data, allowing the integration of diverse types of evidence to prioritize biological associations and demote spurious PPIs [16–18]. To investigate the differential connectivity and relative network occupancy of spliceosomal proteins, we modeled PPIs in the spliceosome as probabilistic events, and built a Bayesian probability model using transitivity and co-expression as supporting evidence (Fig. 1 and Additional file 1). In graph theory, transitivity (also known as clustering coefficient) measures the extent to which a pair of nodes in a network share common interactions with other nodes [19]. This concept was successfully applied to study the organization of other biological networks, such as metabolic networks [20]. In a PPI network, the existence or lack of third-party PPIs can serve as evidence to predict new PPIs or reject false PPIs [21].

Transitivity is appropriate to study a macromolecular complex like the spliceosome, because it rewires PPIs within the boundaries of neighboring proteins. The spliceosome’s structure and function are dictated by the assembly and dissociation of sub-complex units, which are necessary for accurate splicing [2, 3]. It is therefore plausible that spliceosomal proteins remain within the microenvironment of one or a few sub-complexes, so as to maintain the integrity of the entire system.

We made the further assumption that a pair of proteins has to be co-expressed in order to form a PPI. This should ensure a reduction of the number of false positives while emphasizing functionally related PPIs. To this end, we calculated co-expression profiles from microarray data and penalized protein pairs that showed poor co-expression.

**Fig. 2:**
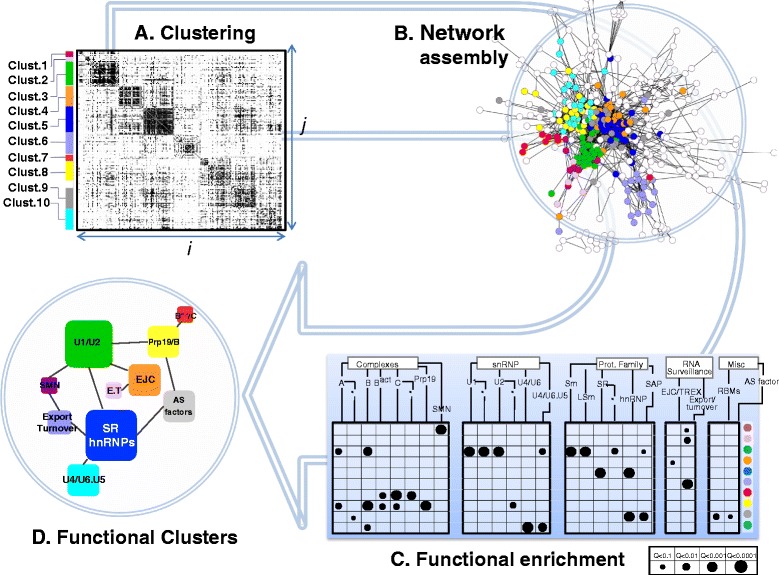
Assembly of the PS network. The flowchart illustrates the identification of functional clusters (FC) of physically/functionally related proteins within the PS network. **a** The adjacency matrix of *P*_*in*_ values for all possible protein pairs was processed with the Hierarchical Clustering algorithm, using Pearson correlation as a distance metric. Clusters were automatically assigned using the Genesis program (every cluster is represented by a different color). **b** Assembly of the PPI network, showing in this example PPIs with cutoff *P*_*in*_ ≥ 0.9. **c** *q*-values resulting from the hypergeometric test to assess the relationship between every cluster and each functional category. Only *q* <0.1 are shown. The size of the bubble is inversely proportional to the *q*-value (bottom right). Functional terms were divided into four categories, and represented as a tree structure. The asterisks indicate groups of proteins that are exclusive to a particular category (for example, C-complex-specific proteins). The colored circles on the right correspond to the clusters identified in A. **d** A network of FCs. FCs are represented as squares labeled with the most significantly enriched functional categories. The square size is proportional to the number of proteins in the FC. Edges are shown for connections with C_IJ_ score >0.2. E.T. = Export and Turnover

To generate this probabilistic model of the spliceosome (Fig. 1 and Additional file 1), we calculated the interaction probability (*P*_*in*_) of 198,135 PPIs formed by 630 splicing-related proteins (Additional file 2: Table S1A) using as evidence 37,231 PPIs and 31,363 co-expression profiles (Additional file 2: Table S1B). We collected these probabilities into an adjacency matrix, showing relationships between all spliceosomal proteins (Fig. 2a, Additional file 2: Table S1C). We used a similar approach as Ravasz *et al.* [20] to visualize associations between topological and functional modules through hierarchical clustering, followed by functional enrichment analysis. Accordingly, we used Pearson correlation coefficients between the binding profiles of each protein (based on *P*_*in*_ scores against the remaining 629 proteins) as a distance metric for hierarchical clustering. We then examined the resulting clusters against a custom list of spliceosome-specific functions (Fig. 2c, Additional file 2: Table S1D) using the hypergeometric test.

We identified 10 different functional clusters (numbered FC1-10) and determined the relative position of the clusters in the spliceosome by scoring cluster-cluster interactions among FCs (Fig. 2d). We refer to the resulting model as the ‘probabilistic spliceosome’ or PS-network. We used this PS-network as a contextual framework to investigate the differential connectivity and relative location of spliceosomal proteins, with a focus on SR proteins and hnRNPs.

### Probabilistic vs. deterministic spliceosome

Y2H datasets are often applied to the construction of deterministic PPI networks (DET), strictly based on direct observations from the data. This approach is subject to multiple errors, due to stochastic undersampling or spurious interactions [22]. One way to reduce false positives in DET networks is to prioritize reproducible PPIs across Y2H experiments [23]. However, among all spliceosomal proteins, PPIs formed by SR proteins and hnRNPs are relatively hard to reproduce (Additional file 3: Table S2A). These selective splicing regulators act by recruiting or blocking spliceosomal sub-complexes (for example, snRNPs) via interactions with proteins or RNA. They also participate in additional processes, such as mRNA export and surveillance or translation regulation [7, 8, 24], and thus they may form transient PPIs with the spliceosome. To circumvent the barrier posed by limited support from Y2H PPIs, we studied the SR protein and hnRNP interactomes through probabilistic modeling.

**Fig. 3:**
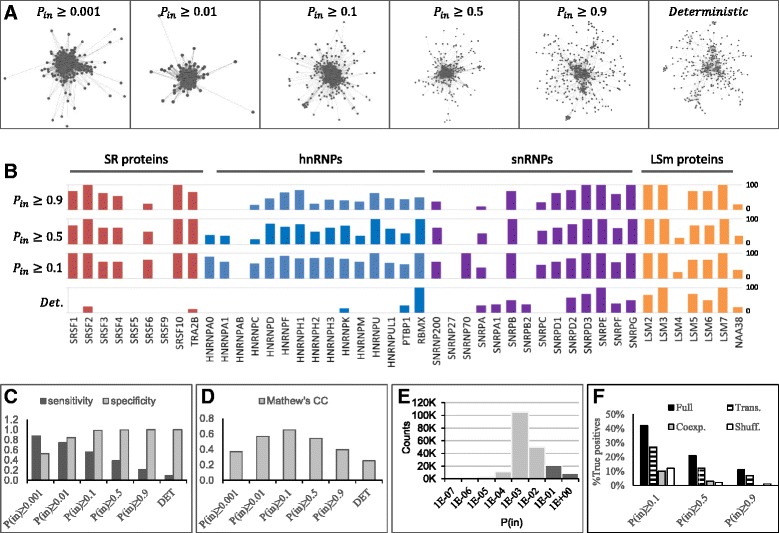
Predictability of the probabilistic spliceosome. **a** PS-networks visualized at different cutoffs: *P*_*in*_ ≥0.001, *P*_*in*_ ≥0.01, *P*_*in*_ ≥0.1, *P*_*in*_ ≥0.5, and *P*_*in*_ ≥0.9 along with a deterministic network of PPIs detected by Y2H. **b-d** Cross-validation results. **b** Predictability by protein family. The height of the column indicates the percent of correctly predicted PPIs for SR proteins (red), hnRNPs (blue), snRNPs (purple), and LSm proteins (yellow). **c** Sensitivity (dark gray) and specificity (light gray). **d** Mathew’s correlation coefficient. **e** Distribution of *P*_*in*_ values in the PS-network. Dark gray indicates values above the threshold *P*_*in*_ ≥0.1. **f** Independent contribution of transitivity and co-expression. The plot shows the percent of correctly predicted PPIs for the full model, using: a combination of transitivity and co-expression (black); transitivity only (dark gray); co-expression (light gray); and as predicted by chance (white)

We conducted a cross-validation analysis to compare the predictability of the PS-network to that of a deterministic network (DET). We used the PPI network from [23] as a test set, and the Human Protein Reference Database (HPRD, [25]) as a training set (see Methods for details). The PS-network was trained as tresholded at *P*_*in*_ ≥0.001, *P*_*in*_ ≥0.01, *P*_*in*_ ≥0.1, *P*_*in*_ ≥0.5, and *P*_*in*_ ≥0.9. Direct PPIs present in the test set were removed from the training set, leaving neighboring PPIs as the sole evidence for probabilistic prediction. We quantified the effect of ignoring direct PPIs for transitivity scoring, and observed that their exclusion left 99.8 % of the estimated *P*_*in*_ probabilities unaffected; only 80/198,135 *P*_*in*_ scores showed residuals ≥0.1 (Additional file 4: Figure S1). Hence, in this work we treat direct and neighboring PPIs equally. Finally, to predict DET PPIs, we counted the net overlap between direct PPIs in the training and test sets. The resulting networks are shown in Fig. 3a.

We tested the ability of the PS- and DET networks to predict transient SR and hnRNP PPIs, as compared to the constitutive interactions of core spliceosomal snRNP and LSm family proteins. Interestingly, SR and hnRNP PPIs could only be predicted using the PS-network. In contrast, core spliceosomal PPIs were detected using either the PS-network or DET network (Fig. 3b, Additional file 3: Table S2B), probably because they are obligatory for spliceosome assembly and therefore easier to detect.

When considering the spliceosome as a whole, probabilistic modeling still outperformed the deterministic approach. For example, the prediction sensitivity of the PS-network was 0.55 using a moderate threshold (*P*_*in*_ ≥0.1) and 0.22 with a stringent threshold (*P*_*in*_ ≥0.9). In contrast, DET network predicted PPIs with sensitivity of 0.1 (Fig. 3c, Additional file 3: Table S2C). The PS-Network predicted up to six times more true positives, with half the number of false negatives compared to DET network (Additional file 3: Table S2C).

For both PS-network and DET network, the prediction specificity was very high (approximately 1), only decreasing to 0.85 and 0.53 when using permissive thresholds of *P*_*in*_ ≥0.01 and *P*_*in*_ ≥0.001, respectively (Fig. 3c, Additional file 3: Table S2C)*.* High specificity is indicative of a low number of false positives. This could be due to the rigorous negative set used in this assay, with pairs of proteins unreachable to each other in a network (see Methods).

We estimated the correlation between the trained and tested classifications using Matthew’s correlation coefficient (MCC), a metric that varies between −1 and 1, 1 being equivalent to a perfect prediction. The PS-network’s top MCC was 0.65 for *P*_*in*_ ≥0.1, whereas DET’s MCC was only 0.25 (Fig. 3d, Additional file 3: Table S2C), demonstrating a gain in predictability by using probabilistic modeling. Based on these results, we set *P*_*in*_ ≥0.1 as the minimal threshold for PPI probabilities, which retained a total of 30,065 PPIs, accounting for less than 5 % of the data variance (Fig. 3e).

In summary, probabilistic modeling through the PS-network is an effective way to predict spliceosomal PPIs. It surpasses deterministic modeling in sensitivity and predictability, and performs with similar specificity. Probabilistic modeling proved especially critical for the study of SR proteins and hnRNPs, for which Y2H data availability is limited.

### Functional clusters represent topologic units

The proteins in the PS-network are not randomly distributed, but instead are clustered in topological modules or FCs (Fig. 2d, Additional file 2: Table S1C). A compacted version of the PS-network (Fig. 2d) shows that early (3 and 8) and late (4, 7, and 10) spliceosomal FCs, as well as pre- (1) and post-splicing FCs (2, 6), are physically separated and resemble functional modules. Of particular interest for this study, FC5 comprises a mixture of nine splicing activators (SRSF1-7, hnRNPU, and RBMX) and five splicing repressors (hnRNAPA1, A2B1, C, H, and SRSF10). In addition FC9 contains a number of activators (hnRNPs F, K, and SRSF9) and repressors (hnRNPL and PTBP1). The activator/repressor activities were assigned based on comprehensive aggregation of literature references derived from the RegRNA database [26] (Additional file 5: Table S3). Although both SR proteins and hnRNPs have been documented to function as activators or repressors depending upon the context, in each individual case one of these two functions occurs much more frequently, allowing for a clear cutoff to distinguish between both groups (Additional file 6: Figure S2).

To examine the topology of the PS-network, we computed the density, modularity, centralization, and average shortest-path length at different *P*_*in*_ thresholds (Additional file 7: Table S4). As *P*_*in*_ increased, the PS network became less dense, more modular, and decentralized. The use of transitivity in our model helped maintain the overall topology by rewiring PPIs only among third-party PPIs. In addition, examination of the independent contributions of transitivity and co-expression to the model revealed that transitivity was the most predictive feature (Fig. 3f). The PS-network at *P*_*in*_ ≥0.9 was topologically identical to DET (Additional file 7: Table S4), indicating that the predicted PPIs are not promiscuous, but reflect selective rewiring of the network. Altogether, we observed that regulatory splicing factors are topologically independent from core spliceosomal proteins, in agreement with the widely accepted notion that the spliceosome is a modular system [2].

### A splicing factor’s activity can be predicted from its connectivity to the spliceosome

**Fig. 4:**
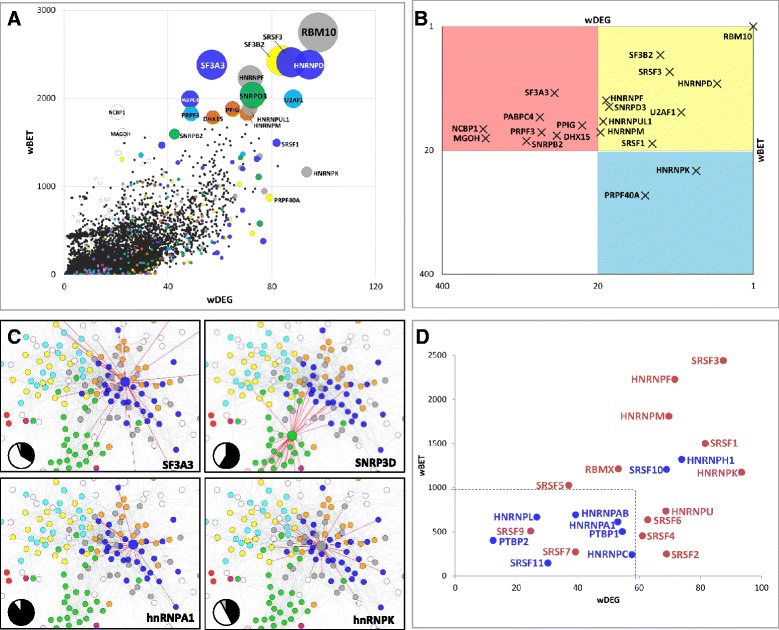
Connectivity of splicing factors to the human spliceosome. **a** Relationship between the Weighted Degree (*wDEG*) and Betweenness (*wBET*) among spliceosomal proteins. Each spliceosomal protein is represented as a bubble. The bubble’s position indicates *wDEG* and *wBET* scores. The size of the bubble denotes *wDEG* or *wBET* statistical significance (−log_10_ of the minimum *q*-value). The color of the bubble specifies the FC to which it belongs (same color code as Fig. 2). White bubbles correspond to unclustered proteins. Black dots represent the *wDEG* and *wBET* scores of 1,000 randomized PS networks. Names of the top 20 statistically significant proteins are shown. For more information, see Additional file 8: Table S5. **b** High-connectivity spliceosomal proteins. Top 20 proteins for wDEG and/or wBET, based on rankings from Additional file 8: Table S5. The yellow square contains proteins in the top 20 for both wDEG and wBET; the blue and red squares contain top scorers for wDEG or wBET, respectively. Both X and Y axes show ranks in logarithmic scale. **c** PPIs at *P*_*in*_ ≥0.9 formed by the designated proteins are shown as red edges (node colors as in Fig. 2). The pie charts indicate the proportion of interactions at *P*_*in*_ ≥0.9 formed between each protein and members of its own cluster (black), other clusters (white), and unclustered proteins (gray). For additional information, see Additional file 10: Figure S3. **d** *wDEG* and *wBET* for splicing activators (red) and repressors (blue) of the SR and hnRNP families, according to annotations in the RegRNA database ([26], Additional file 5: Table S3). The traced square indicates a speculative boundary separating activators from repressors

To identify regulators that play centralizing roles during spliceosome assembly, we computed two standard centrality metrics for every member of the PS-network: ‘Degree’, which is the number of interactions formed by a protein; and ‘Betweenness’, which reflects the extent to which a protein lies between other proteins, acting as a ‘bridge’ in the network. The balance between Degree and Betweenness can shape the modularity of the network, whereby high Degree tends to contribute to intramodular interactions that define biological processes, and high Betweenness contributes to intermodular connections linking different processes [27]. In the case of the spliceosome, we expect that proteins with high Degree are important for complex formation and stabilization, whereas those with high Betweenness control interactions among spliceosomal sub-complexes. We used the *P*_*in*_ values on the edges to compute probability-weighted Degree and Betweenness for every protein in the network. We refer to these as *wDEG* and *wBET*, respectively (Fig. 4a, Additional file 8: Table S5).

A common property of biological (that is, scale-free) networks is the presence of a few nodes with outstanding Degree and/or Betweenness, called hubs, which tend to be encoded by essential genes [28]. To identify hubs that can potentially shape the spliceosome’s modularity, we focused on the top 20 high-connectivity proteins ranked by minimum (wDEG,wBET) *q*-values. Interestingly, many of these proteins are known to play central roles in splicing, and 8/20 have been implicated in diseases, such as cancer (Additional file 9: Table S6). We observed that 10/20 of these proteins were ranked among the top 20 for both *wDEG* and *wBET*, 8/20 were top 20 scorers for *wBET* but not *wDEG*, and only 2/20 scored with high *wDEG* and low *wBET* (Fig. 4b, ranks in Additional file 8: Table S5). This result suggests that spliceosomal hubs often play a dual role of bridging among and within topological modules. For instance, high connectivity proteins tend to form PPIs with multiple FCs, including but not limited to their own FC. Conversely, proteins which scored low in both *wDEG* and *wBET*, such as hnRNP A1, showed skewed interaction profiles: the vast majority of PPIs involving hnRNPA1 were formed with proteins from its own FC (Fig. 4c, Additional file 10: Figure S3).

Of note, seven of the top 20 high-connectivity proteins were SR proteins or hnRNPs, including five known splicing factors. When addressing their centrality, we observed a clear trend: splicing factors labeled activators showed high *wDEG* and *wBET*, whereas repressors scored very low for both (Fig. 4d). With the exception of SRSF10 and hnRNPH1, no splicing repressor scored higher than *wDEG* = 60 and *wBET* = 1,000. Conversely, splicing activators were above these values, with the exception of SRSF7 and SRSF9. Thus, the connectivity of splicing factors to the spliceosome is a strong predictor of their regulatory activity. Moreover, these findings suggest that activators and repressors communicate with the spliceosome’s machinery with different levels of closeness to perform their regulatory tasks.

### IP-MS preparations are enriched in high-probability interactions

To validate the predictability of our model, we performed IP-MS of the prototypical splicing activator SRSF1 and splicing repressor hnRNPA1 (Additional file 11: Figure S4A, B), using T7-tagged constructs that accurately replicate the activities of endogenous SRSF1 and hnRNPA1 (Additional file 11: Figure S4C-M). IP-MS is a useful technique to identify large multimeric protein assemblies. Unlike Y2H, which is designed to capture direct PPIs, IP-MS identifies mixed populations of proteins held in physical proximity through direct or indirect interactions [29].

Because the spliceosome is a ribonucleoprotein complex, we distinguished direct PPIs from PPIs stabilized or mediated by RNA, using differential nuclease treatment [29], followed by IP-MS (Additional file 11: Figure S4N, O). We then classified PPIs as nuclease-resistant (nuc^R^) or nuclease-sensitive (nuc^S^).

**Fig. 5:**
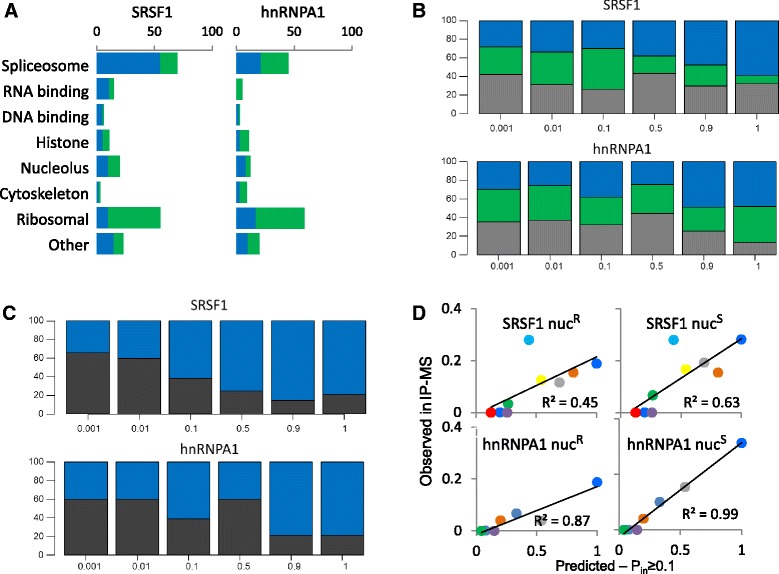
High-probability PPIs enriched by IP-MS. Two splicing factors, SRSF1 and hnRNPA1, were used as baits for IP-MS. The identified proteins were overlaid with the PS-network to identify meaningful patterns of enrichment. **a** The most frequent categories of ligands identified by IP-MS with (blue) or without (green) nuclease treatment. **b** *P*_*in*_ distribution of bait-ligand interactions for SRSF1 and hnRNPA1. The stacked bars illustrate for every P_in_ interval, the proportion of IP-MS ligands recovered with (blue) or without (green) nuclease versus the remaining spliceosomal proteins not identified by IP-MS (gray). The values were normalized by the total number of proteins in every group. **c** Similar to B, comparing P_in_ values from nuclease resistant bait-ligand interactions (blue) to those of ligand-ligand interactions (black). **d** Linear regression between the proportions of observed and expected PPIs (*P*_*in*_ ≥0.1) from each FC. Each dot represents an FC, according to the Fig. 2 color code. Complementary information about this figure is presented in Additional file 13: Figure S5

We identified 203 significantly enriched proteins that co-purified with SRSF1, and 152 with hnRNPA1 (114 and 60, respectively, were nuc^R^) (Additional file 12: Table S7). In all cases, we detected a mixture of spliceosomal and non-spliceosomal proteins, such as histones, ribosomal, cytoskeletal, polynucleotide-binding, and other proteins (Fig. 5a). However, high-probability PPIs where dominated by spliceosomal proteins (Additional file 13: Figure S5A).

**Fig. 6:**
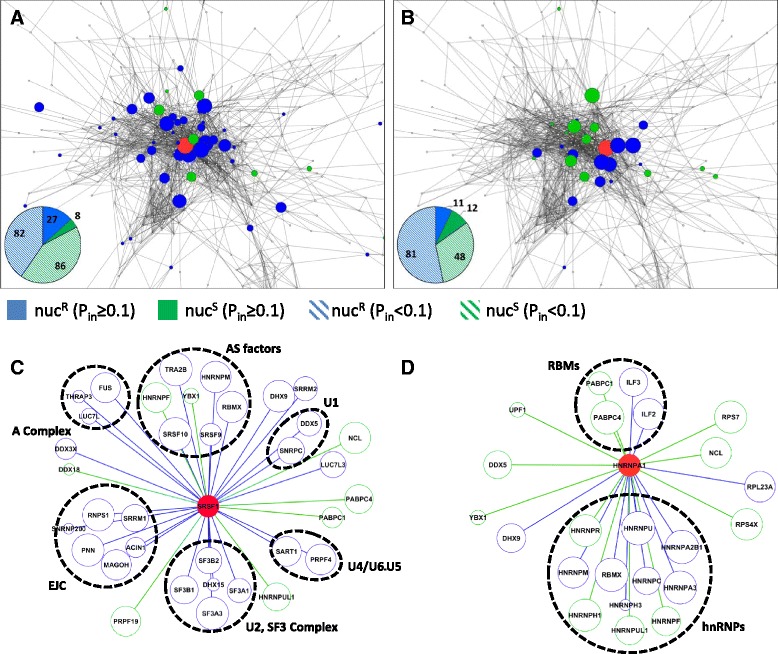
The SRSF1 and hnRNPA1 interactomes. Visualization of the SRSF1 (**a**, **c**) and hnRNPA1 (**b**, **d**) interactomes. (**a**) SRSF1 and (**b**) hnRNPA1 interactomes in the context of the PS network. Nodes representing ligands that form nuc^R^ PPIs with the bait are in blue; nuc^S^ proteins are colored green; IP-MS baits are colored red, and the node sizes are proportional to the *P*_*in*_ scores between each ligand and the bait. Pie charts show the number of nuc^R^ (blue) and nuc^S^ (green) bait-to-ligand interactions with *P*_*in*_ ≥0.1 (dark) and all co-purified proteins (light). (**c**,**d**) High-probability interactions (*P*_*in*_ ≥0.1) detected by IP-MS for (**c**) SRSF1 and (**d**) hnRNPA1. Blue nodes and edges show nuc^R^ PPIs; green nodes and edges show nuc^S^ PPIs; the baits are colored red; functionally related groups of ligands are labeled and indicated with dashed circles

We computed the probability *P*_*in*_ that every identified ligand forms a binary PPI with the baits SRSF1 or hnRNPA1. We observed that IP-MS experiments validated the overall structure of the PS-network, based on the following lines of evidence. First, both nuc^R^ fractions were enriched with high-probability PPIs, as opposed to nuc^S^ fractions that did not show significant deviation from spliceosomal proteins undetectable by IP-MS (Fig. 5b and Additional file 13: Figure S5B). This suggests that nuclease treatment increased the relative proportion of direct PPIs in IP-MS preparations. Second, the average *P*_*in*_ between baits (SRSF1 or hnRNPA1) and ligands (any other protein) was significantly higher than the average *P*_*in*_ between pairs of co-purified ligands (Fig. 5c and Additional file 13: Figure S5C), as expected due to antibody-mediated selective enrichment for bait-ligand PPIs. Third, linear regression between predicted (PS-network) and observed (IP-MS) PPIs in each FC yielded R^2^ scores in the range of 0.45 to 0.99, depending on the bait and the use of nuclease (Fig. 5d). Fourth, co-purified proteins were not scattered throughout the PS-network, but tended to be located in the vicinity of their respective baits (Fig. 6a, b). The average shortest path length between nuc^R^ ligands and the baits was significantly lower compared to IP-MS-undetectable proteins (Additional file 13: Figure S5D). This was not the case for nuc^S^ ligands, implying that only nuc^R^ ligands were predicted by the PS-network as being physically close to the baits SRSF1 and HNRNPA1.

Taken together, these results demonstrate that the PS-network can identify biologically relevant PPIs and categorize spliceosomal proteins. By overlaying the PS-network onto IP-MS data, we uncovered the most plausible interactions, while eliminating contaminants and unspecific PPIs. Thus, we narrowed down SRSF1 and hnRNPA1 IP-MS outputs to generate more specific lists of proteins with high interaction probability. Below we discuss the characteristics of the SRSF1 (Fig. 6a, c) and hnRNPA1 (Fig. 6b, d) interactomes.

### The SRSF1 and hnRNPA1 interactomes

SR proteins and hnRNPs regulate splicing cooperatively or antagonistically, as in the case of the splicing activator SRSF1 and the repressor hnRNPA1 [10, 14]. Here we found that the connectivities of these two proteins to the spliceosome are substantially different. Whereas SRSF1 shows high connectivity to multiple spliceosomal subgroups, the hnRNPA1 interactome is largely restricted (that is, it is mostly composed of additional members of the hnRNP superfamily). In addition, we found that the SRSF1 interactome is rich in direct and RNA-independent PPIs (Fig. 6a, c). In contrast, the hnRNPA1 interactome is smaller and more RNA-dependent (Fig. 6b, d).

#### Multiple connections of SRSF1 to the spliceosome

The largest proportion of SRSF1 ligands detected by IP-MS was, as predicted, dominated by members of FC5 (rich in SR proteins and hnRNPs). Other members of the SRSF1 interactome were previously described, such as the EJC in FC4 [30]. In addition, both IP-MS and our PS-network identified novel interactions of SRSF1 with spliceosomal proteins and complexes. Of particular interest are the SF3a/b proteins, which are components of the U2 snRNP required for early steps in spliceosome assembly. The SF3a/b complex is also the target of many anti-tumor drugs, and among the most highly mutated in various hematological malignancies such as chronic lymphocytic leukemia and myelodysplastic syndromes [31].

We screened the HPRD database for protein complexes containing at least one spliceosomal protein, and counted bait-to-ligand PPIs at *P*_*in*_ ≥0.1 (Additional file 14: Figure S6A). This revealed that out of 144 possible complexes, the SF3a/b complex was the only one predicted to interact with SRSF1 through all of its seven members (0.29 ≥ *P*_*in*_ ≥ to 0.99). In addition, 4/7 members of the SF3a/b complex (SF3A1, SF3A3, SF3B1, SF3B2) were enriched through nuc^R^ IP-MS of SRSF1.

**Fig. 7:**
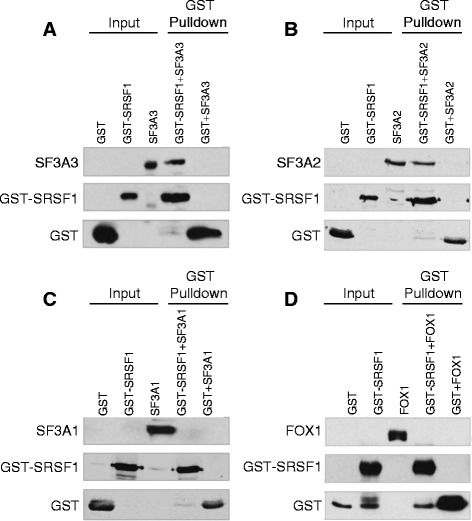
Experimental validation of SRSF1-SF3A PPIs. Purified GST-SRSF1 recombinant protein was incubated with (**a**) His-SF3A3, (**b**) His-SF3A2, (**c**) His-SF3A1, or (**d**) His-FOX1, in the presence of nuclease. GST-SRSF1 was pulled down using glutathione-Sepharose beads, resolved by SDS-PAGE, and interacting partners were detected by anti-His antibody. Purified GST protein was used as a pulldown control

To rigorously validate the direct interaction of SRSF1 with the SF3a/b complex, we tested the binding of three of the IP-MS identified SF3A subunits (SF3A1, SF3A2 and SF3A3) to glutathione-S-transferase (GST)-tagged SRSF1 *in vitro*. GST-SRSF1 interacted efficiently with purified recombinant His-tagged SF3A2 and SF3A3 in the presence of RNase, indicating RNA-independent, direct PPI (Fig. 7). Our predictions were further verified by the absence of interaction between GST-SRSF1 and another splicing regulator, FOX1, which scored very low as an SRSF1-interacting partner (*P*_*in*_ = 0.0002). Although SF3A1 was predicted to interact with SRSF1 and detected as an SRSF1-binding partner in our IP-MS analysis, it did not bind to GST-tagged SRSF1 *in vitro*. Though this indicates an absence of a robust direct interaction between the two proteins, it is also possible that SRSF1 and SF3A1 are weak interactors and require other members of the complex for PPI stability.

In summary, our results indicate that SRSF1 physically interacts with several spliceosomal sub-complexes through RNA-independent interactions. PPIs formed with complexes such as SF3a/b and the EJC are consistent with the fact that SRSF1 is recruited early in spliceosome assembly, yet remains bound throughout the splicing reaction, even after the mRNA is released [2].

#### hnRNPA1 forms RNA-dependent regulatory interactions

Most PPIs formed by hnRNPA1 were with other hnRNP proteins (Fig. 6d, Additional file 14: Figure S6B). A minority of PPIs were nuc^R^, mostly from FC5 (hnRNPs A2B1, A3, C, U, and RBMX) which also contains hnRNPA1 itself. In contrast, nuc^S^ PPIs localized mostly to FC9 (hnRNPs A0, F, H3, K, L, and UL1), suggesting that within FCs, hnRNPs are physically bound, whereas across FCs, they interact through binding the same mRNA.

To investigate the interplay between PPIs and regulatory interactions among hnRNPs, we utilized a list of frequently co-occurring hnRNP binding sites in pairs of intronic regions associated with alternative splicing [32]. Strikingly, we observed that the vast majority of regulatory interactions among hnRNPs involved members across different clusters, rather than members of the same cluster (Additional file 15: Figure S7A). Using Fisher’s test, we estimated that the probability of such a distribution to occur by chance is approximately 10^−7^. Taking into consideration the information about nuclease sensitivity obtained by IP-MS, we then generated a combined picture of PPIs, regulatory interactions, and RNA dependence (Additional file 15: Figure S7B). We observed a clear pattern in which hnRNPA1 interacted with proteins from its own group (FC5) through physical contact in an RNA-independent way, albeit without forming regulatory collaborations. Conversely, hnRNPA1 connected with members of another group (FC9) by forming multiple co-regulatory interactions, but no direct, RNA-independent physical contact.

These results suggest that the partition of hnRNPs into two separate domains of the spliceosome may be important for their function in splicing regulation (Additional file 15: Figure S7C). Furthermore, our data on hnRNPA1 support a previously suggested regulatory mechanism of hnRNP-mediated bridging, and helps to explain why hnRNPs are so highly cooperative and often interchangeable [9, 11].

## Discussion

The mechanism of splicing has been extensively studied; previous work has largely focused on constitutive elements necessary for precise splicing [1, 23, 33, 34] or on the discovery of alternative exons regulated by individual splicing factors [9–12, 14]. Here we emphasized the contextual connectivity of splicing factors in the spliceosome, and their relationships with other spliceosomal proteins.

We used Bayesian probability to predict PPIs by interrogating different data types (for example, Spliceosome DB, KEGG, regRNA) and many literature resources to construct a probabilistic model of the human spliceosome. The posterior probability of true PPIs was computed using the connectivity of PPI modules as evidence, and fine-tuned by orthogonal information obtained from gene-expression microarrays. The resulting PS-network was essential to uncover a large number of novel PPIs among SR proteins and hnRNPs (Fig. 3b). In contrast, the number of newly discovered PPIs in the subset of core spliceosomal proteins was small. This distinction between selective and core spliceosomal proteins may be due to differences in their functional properties. For instance, Papasaikas *et al.* [35] recently reported a functional splicing network integrating knockdown profiles for all spliceosomal proteins. A key observation in this study was that core spliceosomal proteins show outstanding functional connectivity, compared to selective splicing regulators, including SR proteins and hnRNPs. This finding reinforces the notion that the functional selectivity of regulatory splicing factors may negatively affect the reproducibility of PPI detection through Y2H.

Analysis of the PS-network revealed a trend whereby splicing activators engage in a relatively large number of PPIs with other proteins in the spliceosome, perhaps playing an active role in recruiting spliceosomal proteins. In contrast, repressors display fewer PPIs (as was the case for hnRNPA1), suggesting that they predominantly affect splicing by steric interference through RNA binding. IP-MS experiments confirmed these rules for the prototypical splicing factors SRSF1 and hnRNPA1. In both cases, IP-MS fractions were enriched in high-probability interactions, as predicted by our model. This was especially noticeable for the samples treated with nuclease.

SRSF1 formed multiple nuc^R^ PPIs with multiple FCs. Among the top-scoring ones, we observed components of the SF3a/b complexes, which are essential for spliceosome assembly, and tether the U2 snRNP to the pre-mRNA, contributing to branch-site recognition [33]. Interestingly, SR proteins were previously observed to promote the recruitment of U2 snRNP to the pre-mRNA branch site [36]. In addition, a study of the protein composition of the 17*S* U2 snRNP revealed that SRSF1 is present in immunopurified complexes containing SF3a66 [34]. Our analysis here predicted that all members of the SF3a/b complex bind SRSF1, with probabilities in the range of 0.29 to 0.99. The fact that all SF3 subunits are predicted to bind to SRSF1 with high probability is not surprising, given that interactions among the SF3 subunits are strong, not only physically, but also functionally [35]. We validated these interactions in cells (SF3A1, SF3A3, SF3B1, and SF3B2 were significantly enriched by IP-MS with nuclease treatment), and *in vitro* (SF3A2 and SF3A3 were validated through GST-pull-downs). These results indicate that our Bayesian model faithfully predicts PPIs that can be experimentally validated. In addition, these novel PPIs are interesting for their potential implications in cancer. As both SRSF1 and SF3B1 are misregulated in various human tumors [31, 37], and as SRSF1 can transform epithelial cells *in vivo* [38], it would be of interest to determine if altering the SRSF1 and SF3-mediated recruitment of the U2 snRNP plays a role in tumorigenesis.

In contrast to SRSF1, hnRNPA1 displays weaker and less widespread interactions with the spliceosome. Most high-probability hnRNPA1 PPIs were nuclease-sensitive, and as predicted, most IP-MS-confirmed PPIs involved additional members of the hnRNP superfamily. Combining our data with previously reported regulatory interactions [32], we demonstrate that hnRNPs are distributed in at least two highly interconnected clusters, forming regulatory collaborations. Our data strengthen the notion that hnRNPs collaborate through RNA binding. A recent study [9] showed that a group of six hnRNPs (A1, A2B1, H1, F, M, and U) are highly cooperative in regulating alternative splicing. Using CLIP-seq and microarray analyses, the authors observed robust co-regulation between pairs of hnRNPs. Our analysis not only supports this observation, but further indicates that many of these interactions occur between hnRNPs that belong to different clusters, such as hnRNPs A1 (FC5) and M (FC4) or hnRNPs F (FC9) and U or A2/B1 (FC5).

One possible reason for this disparity stems from the inherent differences between activators and repressors as biochemical entities. Splicing activators may modulate spliceosome assembly through the formation of multiple PPIs, and in this way ensure *bona-fide* splice site recognition and exon inclusion. In contrast, repressors may form fewer interactions to block the spliceosome’s attempts to recognize and eventually include an exon. Hence, whereas activators may coordinate and enhance the connectivity of spliceosomal sub-complexes, in the case of repressors it may be sufficient to bind specifically to cognate motifs on the RNA and block spliceosome assembly or activity. The functionality of SR proteins and hnRNPs is evolutionarily conserved [39] and their selective roles as activators or repressors has been documented in numerous studies, ranging from cell-free splicing to minigene transfection experiments to high-throughput analyses (Additional file 5: Table S3 and Additional file 6: Figure S2). Some of these proteins, like SRSF1 and hnRNPA1, have been intensely studied, whereas others have only recently been functionally characterized (for example, SRSF10 and hnRNPU). Previous work has demonstrated the complexity of splicing regulation by showing that a given SR protein or hnRNP can function as both activator and repressor, depending on the sequence-specific and positional context [40, 41]. In these studies, tethering SR proteins (or hnRNPs) upstream or downstream of the 5′SS [40], or changing the position of an SR protein binding motif along the exon [41] resulted in alteration of the regulatory activity of splicing activators to repressors or vice versa. Thus, consistent with annotations in RegRNA, under certain conditions splicing activators and repressors can switch their activities. The generality of this duality remains to be determined, for example, by integrating multiple RNA-seq datasets to assess the reproducibility of effects on specific splicing targets, while neutralizing indirect or sporadic splicing changes.

## Conclusion

This work summarizes our initial attempt to combine public data with our own IP-MS data to understand structure/function relationships in the human spliceosome. Our network-based approach utilized data integration to understand the contribution of individual proteins to the spliceosome as a whole. We characterized key splicing factors, expanding the knowledge about their regulatory mechanisms and discovering new PPIs with therapeutic potential. Altogether, this demonstrates the usefulness of our approach to explore and characterize the mechanistic principles governing complex biological machines.

## Methods

### Datasets

A total of 630 spliceosomal and splicing-related proteins were collected from the Spliceosome DB [42], KEGG [43], and other literature references [1, 23] (Additional file 2: Table S1A). This compendium comprises functionally confirmed spliceosomal proteins, but also proteins related to other RNA-maturation processes, such as mRNA surveillance, export, capping, and polyadenylation. We included the latter proteins because they typically co-purify with the spliceosome [1, 23] and are functionally associated or coupled with splicing [7, 8, 44]. Throughout the manuscript, we consider this extended set of proteins as ‘spliceosomal proteins’. A total of 37,231 PPIs formed by these proteins were extracted from HPRD [25] and Hegele *et al.* [23]. In total, 31,363 co-expression profiles between mRNAs coding for these proteins and PPI partners were collected from the Human U133A/GNF1H microarray dataset [45] (Additional file 2: Table S1B).

### Probabilistic reconstruction of the spliceosome

We developed a Bayesian model to estimate the posterior probability that any given pair of proteins in the spliceosome forms a binary PPI. Our model is based on the principle of transitivity (*T*), which states that a binary interaction between two proteins is more likely if they share a substantial number of interacting partners [19]. The model also incorporates microarray co-expression profiles (*C*), to prioritize genuine from spurious PPIs.

We treated *T* and *C* as two independent variables, and computed conditional probabilities using HPRD data to represent binding instances in a true PPI network (*e* = 1), (Additional file 16: Figure S8A, C) and a ‘decoy’ PPI network to represent non-binding instances (*e* = 0) (Additional file 16: Figure S8B, D). The model is fully explained in Additional file 1.

### Data clustering

Pearson correlation coefficients between all pairs of proteins in the PS-network were calculated using as an input the adjacency matrix of PPI probabilities (*P*_*in*_) (Additional file 2: Table S1C). As a result a second matrix (distance matrix) was obtained, describing the extent of similarity between protein pairs in terms of their binding preferences. Subsequently, this matrix was clustered using averaged hierarchical clustering on both columns and rows. All the clusters and distance matrices were derived using the Genesis program [46, 47].

### Hypergeometric test

To dissect the functionality of every cluster, we performed enrichment analysis using the hypergeometric test. We tested every cluster against a custom list of spliceosome-specific functions, similar to gene ontologies or gene lists (Additional file 2: Table S1D). This list was constructed based on information from Spliceosome DB [42] and KEGG [43], allowing us to explore splicing-related functions in greater detail than offered by standard tools.

This test attempts to reject the null hypothesis that the overlap between two categorical groups (a cluster and a biological function) is due to chance. We used the hypergeometric test to compute exact *P* values for the enrichment of functional terms (that is, ontologies) in the network clusters, according to the formula:

$$ HG\left(b;N,B,n\right)=\frac{\left(\begin{array}{c}\hfill n\hfill \\ {}\hfill b\hfill \end{array}\right)\left(\begin{array}{c}\hfill N-n\hfill \\ {}\hfill B-b\hfill \end{array}\right)}{\left(\begin{array}{c}\hfill N\hfill \\ {}\hfill B\hfill \end{array}\right)} $$

Where ‘*N*’ is the total number proteins in the network, ‘*B*’ is the number of proteins that belong to a given functional term, ‘*b*’ is the number of proteins that belong to a certain cluster, and ‘*n*’ is the number of proteins that belong both to a cluster and a functional term. Finally, we applied the false discovery rate (FDR) procedure to adjust the resulting *P* values.

### Network layout

Network topologies were generated using Cytoscape [48]. This implements a force-directed algorithm that sets the positions of the nodes by minimizing a function that mimics physical repulsion between nodes. Accordingly, the positions of the nodes depend on the length and number of edges. The edge length is inversely proportional to the value of *P*_*in*_; as a result, the layout of the network is such that densely connected proteins appear in the center, whereas low-degree proteins are more peripheral. We used *P*_*in*_ ≥0.1, *P*_*in*_ ≥0.5, and *P*_*in*_ ≥0.9 for visualization. The corresponding thresholds are stated in each figure legend.

### Cluster-cluster interactions

The connectivity C_IJ_ between two clusters I and J was calculated as the sum of the interaction probabilities between all protein pairs spanning FCs I and J, normalized by the sum of probabilities connecting I and J to all possible FCs in the network.

$$ {C}_{IJ}=\frac{{\displaystyle {\sum}_{i\in I,j\in J}}{P}_{Ij}}{{\displaystyle {\sum}_{i\in I,n\in N}}{P}_{in}+{\displaystyle {\sum}_{j\in J,n\in N}}{P}_{jn}-{\displaystyle {\sum}_{i\in I,j\in J}}{P}_{ij}} $$

### Cross-validation assay

We tested the predictability of our model using the network derived from [23] (test set). As a training set we used PPIs from HPRD. To train the PS-network, we omitted PPIs in HPRD that were also present in [23]. These were set aside and used for deterministic predictions. We considered as positive PPIs any pair of proteins i and j from the test set with evidence of forming direct PPIs. The total number of positive PPIs was 601. Negative PPIs were protein pairs from the test network whose shortest path length was L(i,j) ➔ ∞. In this way, both proteins are unreachable through any path in the network, and are not expected to interact directly or indirectly. The number of negative PPIs was 1524. Consequently, true positives (TP) were defined as all successfully predicted PPIs using the training set, whereas false negatives (FN) were PPIs that failed to be predicted. Similarly, false positives (FP) were positively predicted PPIs from the negative set, and finally, true negatives (TN) were undetected protein pairs from the negative set.

To quantify the predictive performance we computed the following metrics: (1) sensitivity (also known as true positive rate) and (2) specificity (also known as true negative rate), both of which return values between 0 and 1. A value of 1 means that there are no false positives/negatives; 0.5 means that there are as many false positives/negatives as true positives/negatives; 0 means that no true positives/negatives were detected. In addition, we reported (3) Matthew’s Correlation Coefficient, which measure the extent of agreement between observed and predicted binary classifications. It returns values between −1 and +1. A coefficient of +1 represents a perfect prediction, 0 no better than random prediction, and −1 indicates total disagreement between prediction and observation:

1. $$ sensitivity = \raisebox{1ex}{$TP$}\!\left/ \!\raisebox{-1ex}{$\left(TP+FN\right)$}\right. $$
2. $$ specificity = \raisebox{1ex}{$TN$}\!\left/ \!\raisebox{-1ex}{$\left(TN+FP\right)$}\right. $$
3. $$ Mathe{w}^{\hbox{'}}s\ CC = \frac{\left(TP*TN\right)-\left(FP*FN\right)}{\sqrt{\left(TP+FP\right)\left(TP+FN\right)\left(TN+FP\right)\left(TN+FN\right)}} $$

### Topological network measures

#### Density

The ratio between existing and potential edges. It is a measure of how heavily interconnected the nodes in a network are.

#### Average shortest path length

The average number of steps along the shortest path, for all possible pairs of network nodes. It is a measure of the closeness between the nodes.

#### Modularity

A measure of how strongly the network is divided into communities of highly interconnected nodes. It is measured as the fraction of edges that fall within given communities minus the expected fraction if the edges were distributed at random.

#### Centralization

Networks whose topologies resemble a star have centralization close to 1, whereas decentralized networks are characterized by having centralization close to 0. This is a measure of how evenly distributed the edge density of the network is.

### Network analysis

Network density, shortest path length, and modularity were calculated using the iGraph R package ([49]). Weighted Degree (*wDEG*) and Betweenness (*wBET*) were calculated using the Tnet R Package [50]. Briefly, the *wDEG* was calculated as the sum of the probability of the edges connecting protein i to any protein j.

$$ wDE{G}_i={\displaystyle \sum_{i\ne j\in N}}{P}_{ij} $$

*WBET* of protein *i* in a network N is defined as:

$$ wBE{T}_i={\displaystyle \sum_{s\ne i\ne t\in N}}\frac{W{L}_{st}(i)}{W{L}_{st}} $$

Where *WL*_*st*_ is the probability-weighted path length from node *s* to node *t*, and W*L*_*st*_*(i)* is the number of those paths passing through *i*. The minimal W*L*_*st*_ for every protein pair is considered as the weighted shortest path length.

To identify proteins with statistically significant *wDEG* or *wBET*, we estimated *q*-values by comparing *wDEG* or *wBET* scores to the distribution of 1,000 randomized networks generated through the Erdős–Rényi procedure [51], followed by FDR correction.

### Complex analysis

Annotated protein complexes were downloaded from HPRD and searched against our list of 630 spliceosomal proteins. A total of 144 complexes containing spliceosomal proteins were selected and tested for whether they form *P*_*in*_ ≥0.1 PPIs with either SRSF1 or hnRNPA1, were detected by IP-MS, and survived nuclease treatment.

### Plasmids

Construction of MSCV-TT-T7SRSF1 was previously described [52]. MSCV-TT-T7hnRNP A1 was generated by subcloning the hnRNPA1 open reading frame (ORF) from pCG-hnRNPA1 [53] downstream of the tetracycline-responsive promoter (TRE-tight) in the retroviral MSCV vector (kindly provided by Scott Lowe). The GST-SRSF1 bacterial expression plasmid was generated by subcloning the SRSF1 open reading frame from pCG-SRSF1 in the pGEX-3X vector (GE Lifesciences). His-tagged recombinant SF3A (SF3A1, SF3A2, SF3A3) plasmids were generated by sub-cloning the SF3A ORFs from pBLUESCRIPT plasmids generously provided by Robin Reed into the pET28a (+) vector. His-tagged FOX1 was generated by sub-cloning the FOX1 ORF [54] into the pET28a (+) vector.

### Cell culture and cell lines

All cells were grown in DMEM-Complete (Gibco) supplemented with 10 % (v/v) fetal bovine serum (FBS, Thermo), 100 U/mL penicillin (Gibco), and 1,000 μg/mL streptomycin (Gibco). Lentiviruses were generated as described [37]. To generate Doxycycline-inducible cell lines, HeLa Tet-on Advanced cells (Clontech) were infected for 48 h, allowed to recover for an additional 24 h, and selected with the appropriate antibiotic.

To induce HeLa TT-T7SRSF1 and TT-T7hnRNPA1 cells, doxycycline was added to the cells at a concentration in the range of 0.01 to 10 μg/mL for 24 to 48 h, depending on the assay. For affinity purifications and immunofluorescence, TT-T7SRSF1 cells were induced with 0.1 μg/mL, and TT-T7hnRNPA1 cells with 0.5 μg/mL doxycycline for 36 h. These values were determined by western blotting (Additional file 11: Figure S4A, B) as resulting in overexpression of the T7-tagged protein within two-fold compared to the endogenous counterpart, and at the same time not resulting in any visible cell death.

### Gel electrophoresis and immunoblotting

Lysates were separated by SDS-PAGE and probed with the indicated antibodies. Primary antibodies against the following proteins/epitopes were used: T7 (1:500), SRSF1 (AK-96, 1:500), hnRNPA1 (AK-55, 1:50). HRP-conjugated goat anti-mouse or anti-rabbit (Biorad, 1:10,000) antibodies were used for chemiluminescent detection [52]. AK-96 and AK-55 were previously described [55]. Silver-stained gels were stained with a SilverQuest kit (Invitrogen), following the manufacturer’s instructions.

### Fluorescence microscopy and immunolocalization

Cells were plated in Fisher 6-well chamber slides at a density of 20,000 cells/well. Twenty-four hours later, doxycycline was added and the cells were incubated for an additional 36 h. Indirect immunofluorescence was modified from [56]. Cells were incubated with the appropriate fluorescence-conjugated secondary antibody (Invitrogen). 4′,6-diamidino-2-phenylindole (DAPI; Boehringer-Mannheim) was used to stain the nuclei. Microscopy was performed on a Zeiss Axiovert 200 M, using Axiovision 4.4 and the ApoTome imaging system.

### Preparation of cell extracts

For general protein analysis of whole-cell lysates, cells were lysed in RIPA Buffer (150 mM NaCl, 1 % (v/v) NP-40, 0.5 % (w/v) deoxycholic acid, 0.1 % (w/v) sodium dodecyl sulfate (SDS), 50 mM Tris pH 8.0) plus Roche Protease Inhibitor Cocktail EDTA-free. Cell lysis followed by immunoprecipitation was performed as in [52]. Four 15-cm plates were used for each condition. Where appropriate, nuclease was added (1 U/mL RNase A, 40 U/mL RNase T1, 500 U/mL Benzonase, plus 2 mM MgCl_2_) for 30 min, prior to immunoprecipitation.

### Immunoprecipitation of protein complexes

Dynabeads Protein G (Invitrogen) was used for all IPs, according to the manufacturer’s instructions. For all immunoprecipitations, lysates were incubated with immobilized antibodies while rotating for 1 h at 4 °C and washed five times with 1 mL of Lysis Buffer (0.05-0.5 % (v/v) NP-40, 100–500 mM NaCl, 50 mM Tris, pH 7.4, 1 mM DTT). For mass spectrometry, peptides were eluted by on-bead digestion [57] and samples were prepared as in [52].

### Multidimensional chromatography and tandem mass spectrometry

Following immunoprecipitation and on-bead trypsin digestion, samples were analyzed by on-line 7-step MudPIT HPLC, and LTQ mass spectrometry.

Briefly, peptide mixtures were analyzed by MudPIT through a protocol adapted from [58] using a two-dimensional vented volume setup with a Proxeon nano-flow HPLC pump [59]. Triphasic MudPIT columns were packed in-house with alternating Aqua C-18 reverse phase material and Luna strong cation exchange material (Phenomenex). HPLC runs were automated following a protocol adapted from [60] with a constant flow-rate of 300 nL/min. Following separation on the MudPIT column, peptides eluted from the microcapillary fritless column were directly electrosprayed into a linear ion trap (LTQ) mass spectrometer (Thermo Finnigan). A cycle of one full-scan mass spectrum (400–1700 m/z) was acquired with enhanced scan rate, followed by six data-dependent MS/MS spectra at a 35 % normalized collision energy. Dynamic exclusion lists of 500 spectra were set to exclude peptides for a duration of 90 s. Mass-spectrometer scan functions were controlled by the Xcalibur data system (Thermo Finnigan) and data were processed with MASCOT Distiller (Matrix Science) using the default parameters for ion-trap data analysis. LTQ MS/MS spectra were searched with MASCOT version 2.2.04 against the human IPI non-redundant database (version 3.35). The number of hits identified by Mascot in every replicate is shown in Additional file 17: Table S8. The MS dataset is available at [61].

### Identification of proteins over-represented upon doxycycline treatment

Each IP-MS experiment was carried out in duplicate (Additional file 17: Table S8). The overlap between the duplicates was approximately 50 % for the Dox^+^ and approximately 30 % for the Dox^−^, for both SRSF1 and hnRNPA1 with nuclease versus without nuclease.

The enrichment of every protein identified upon IP-MS of SRSF1 or hnRNPA1 was calculated as follows:

$$ \mathrm{logE}={ \log}_2\left(\frac{\left({\mathrm{P}}_{\mathrm{DOX}+}\right)}{\left({\mathrm{P}}_{\mathrm{DOX}-}+1\right)}\right) $$

Where P_Dox+_ was the number of unique peptide counts per protein identified at >95 % confidence in the IP experiment, and P_Dox-_ was the corresponding number of peptides identified without doxycycline induction. To account for cases in which the protein was below the detection sensibility in Dox^−^ but not Dox^+^, we added a pseudo-count to the denominator. We set a cutoff at logE = 1 as a threshold. In this way, we ensured that all the selected proteins would be represented by a two-fold ratio and at least three peptides.

### Purification of recombinant proteins

GST-SRSF1 was expressed in E. coli BL21(DE3)pLysS strain by induction with 0.5 mM IPTG overnight at 18 °C and purified using glutathione-Sepharose beads (GE Healthcare). His-tagged SF3A1, SF3A2, SF3A3, and FOX1 were similarly expressed and induced, but purified using Ni-NTA Agarose (Qiagen).

### GST pulldown

Purified GST-SRSF1 (200 pM) was incubated with His-tagged recombinant proteins (200 pM) (His-SF3A1, His-SF3A2, His-SF3A3, His-FOX1) and 50 μL glutathione-Sepharose beads in 300 μL of pull-down buffer (20 mM HEPES, pH 7.5, 100–500 mM NaCl, 0.5 mM EDTA and 0.1-0.5 % (v/v) NP-40) for 2 h at 4 °C in the presence of nuclease (1 U/mL RNase A, 40 U/mL RNase T1). The resin was washed three times with 500 μL of pull-down buffer. Proteins were eluted with 40 μL 1X Laemmli buffer, resolved by SDS-PAGE probed with anti-GST and anti-His antibodies and analyzed on an Odyssey infrared-imaging system (LI-COR Biosciences).

## Additional files

Additional file 1:
**Supplementary Methods section.**
Additional file 2: Table S1. Components of the PS-network. (A) Proteins that constitute the nodes in the PS-network and sources linking them to the spliceosome. (B) PPIs used to train the Bayesian model with their corresponding co-expression levels (Pearson correlation). (C) PPI probability adjacency matrix representing the edges of the PS-network. The colors correspond to the clusters in Fig. 2. (D) Functional categories of spliceosomal and splicing-related proteins. Additional file 3: Table S2. Annotated spliceosomal PPIs and predictability of the PS-network. (A) Reproducibility of PPIs annotated in HPRD and Hegele *et al.* for each functional category of spliceosomal proteins. The column ‘#proteins’ indicates the total number of proteins in every group. ‘Intersection’ is the number of overlapping PPIs between both datasets, and ‘ratio’ is the ‘intersection’ normalized by ‘#proteins’. The functional groups are ranked by their ‘ratio’. SR proteins and hnRNPs are highlighted in gray. (B) Number of PPIs per protein predicted by the PS-network versus deterministic modeling. The left side of the table shows the absolute number of PPI predicted for every protein in the test set. The right side shows the percent of the total PPIs per protein. (C) Predictability metrics of the PS-network and deterministic network. Additional file 4: Figure S1. Effect of direct PPIs on the PS-network. The effect of excluding direct PPI annotations, as opposed to treating them equally to neighboring interactions, for transitivity estimation based on Y2H data. We computed *P*_*in*_ scores with direct PPIs (Full model) or without (Partial model). (A) Correlation between *P*_*in*_ using vs. ignoring direct PPIs. (B) Residual *P*_*in*_ between Full vs. Partial models. (C) Histogram of the distribution of residual scores. Additional file 5: Table S3. References for splicing activator or repressor activities of SR proteins and hnRNPs . Following annotations in RegRNA [26] and additional literature sources ESE (exonic splicing enhancer), ESS (exonic splicing silencer), ISE (intronic splicing enhancer), ISS (intronic splicing silencer). Additional file 6: Figure S2. Classification of SR proteins and hnRNPs into activators and repressors. Based on annotations from RegRNA ([26], Additional file 5: Table S3). The bar plot shows the number of literature references supporting a splicing factor’s activity as activator (red) or repressor (blue). The labels activator or repressors were assigned based on the best supported function of each protein. Labels are shown on the left side of the chart. Additional file 7: Table S4. Topological metrics of the PS-network and deterministic network. Additional file 8: Table S5. Centrality metrics of spliceosomal proteins. Additional file 9: Table S6. Involvement of high-connectivity spliceosomal proteins in human disease. Additional file 10: Figure S3. PPIs formed by top-centrality spliceosomal proteins. See legend from Fig. 4b. Additional file 11: Figure S4. Immunoprecipitation of SRSF1 and hnRPA1. (A, B) Induced expression of SRSF1 and hnRNPA1 in HeLa cells. (A) HeLa T7-SRSF1 and (B) HeLa T7-hnRNPA1 cells were induced for 36 h with increasing Dox concentrations (from 0.01 to 10.0 μg/mL). Whole-cell lysates were analyzed by western blotting. T7-tagged proteins are marked with an arrow to distinguish them from endogenous proteins. β-catenin was used as a loading control. All cell lines showed a linear response to Dox when probed with endogenous-protein and T7-tag antibodies, although the levels of the T7-tagged splicing factors varied between cell lines. We used 36 h of induction at 0.1 μg/mL Dox for T7-SRSF1, and 0.5 μg/mL Dox for hnRNPA1. (C-M) Cellular localization of induced HeLa TT-SRSF1 and hnRNP A1 is consistent with expression of the endogenous proteins. (C) Indirect immunofluorescence of endogenous (uninduced) SRSF1, (D-F) induced T7-tagged SRSF1 and (G-I) hnRNP A1. (J-M) Co-staining of endogenous SRSF1 (AK-96) and induced T7-SRSF1. Cells were induced with Dox for 36 h at 0.1 μg/mL (SRSF1) and 0.5 μg/mL (hnRNP A1). DNA was stained with DAPI. (N, O) T7-SRSF1 immunoprecipitation (IP) with nuclease treatment: (N) Whole-cell lysates of HeLa TT-SRSF1 cells, with and without nuclease treatment. Cells were induced with Dox for 36 h at 0.1 μg/mL. Nuclease consists of RNases A and T1, plus Benzonase. (Middle) Co-IP of T7-SRSF1 in the presence or absence of nuclease. Co-IP was performed in the presence of 200 mM NaCl. (O) Silver stain of immunoprecipitates. Additional file 12: Table S7. PPIs detected by IP-MS and estimated through the PS-network. For (A) SRSF1 and (B) hnRNPA1 against other proteins. Additional file 13: Figure S5. High-probability PPIs enriched by IP-MS (continued from Fig. 5). (A) The colored tables show the distribution of *P*_*in*_ scores for SRSF1 and hnRNPA1 co-purified proteins. These were divided into three categories, depending on whether they are spliceosomal proteins assigned to specific FCs (spliceosomal, clustered) unassigned to FCs (spliceosomal, non-clustered) or non-spliceosomal. The average *P*_*in*_ scores for each category are shown at the bottom. (B) Beanplot showing *P*_*in*_ distribution of bait-ligand interactions for SRSF1 and hnRNPA1 for IP-MS with (blue) or without (green) nuclease, and for the remaining spliceosomal proteins not identified by IP-MS (gray). Red lines indicate mean values. (C) Similar to B, comparing P_in_ values from nuclease resistant bait-ligand interactions (blue) to those of ligand-ligand interactions (black). (D) Boxplot showing the distribution of weighted shortest path lengths for either SRSF1 or hnRNPA1 co-purified proteins with (blue) or without (green) nuclease treatment. The remaining spliceosomal proteins that failed to be purified are marked in gray. Wilcoxon test *P* values are shown in B-D represented by stars as follows: **P* <0.05, ***P* <0.01, ****P* <10^−3^, and *****P* <10^−5^. Additional file 14: Figure S6. Complex-composition analysis. Distribution of predicted and co-purified PPIs for (A) SRSF1 and (B) hnRNPA1 among protein complexes annotated in HPRD. Additional file 15: Figure S7. Physical and regulatory interactions among hnRNPs are mutually exclusive. (A) Regulatory interactions among hnRNPs. The node color indicates the cluster to which each protein belongs, according to the code in Fig. 3d. The edges represent regulatory interactions, as reported by Ke and Chasin [32]. (B) Patterns of interaction between hnRNPA1 and other members of the hnRNP superfamily. Solid edges denote PPIs resistant to nuclease treatment. Dashed edges indicate nuclease-sensitive PPIs. Red edges denote regulatory interactions. The numbers along the edges indicate *PI* values. (C) Model summarizing co-regulation among hnRNPs. hnRNP pairs from different spliceosomal blocks usually cooperate to regulate alternative splicing. In particular, most proteins belong either to FC5 (blue) or FC9 (gray). Additional file 16: Figure S8. Conditional probability models. Cumulative distributions for transitivity (A) were calculated using HPRD to represent true binding instances. (B) A similar distribution was derived from a ‘decoy’ HPRD (dHPRD), to represent non-binding instances. In the same way, we generated true (C) and decoy (D) co-expression distributions, by combining both HPRD and dHPRD with the Human U133A/GNF1H microarray dataset. Additional file 17: Table S8 Summary of MASCOT results. Number of significant peptides detected.
