# Supplementary material for: Differential connectivity of splicing activators and repressors to the human spliceosome
Source: Genome Biol. 2015 Jun 6;16(1):119. doi: 10.1186/s13059-015-0682-5 (PMC4502471; doi:10.1186/s13059-015-0682-5)
Supplement: Additional file 1: — Supplementary Methods section. [file 13059_2015_682_MOESM1_ESM.pdf]

Probabilistic reconstruction of the spliceosome

We developed a probabilistic model that utilizes the Bayesian rules of inference to estimate the posterior probability that any given pair of proteins in the spliceosome forms a binary (or direct) PPI. By definition, the posterior probability is the revised likelihood of an event occurring, after taking into consideration supporting evidence and prior information. Their choice depends on the underlying assumptions, relevant data, and the queries made about the system.

There are numerous methods to predict PPIs [1]. We based our model on the Bayesian setup proposed by Asthana et al. [2] due to its flexibility for new types of supporting evidence. Asthana's model utilizes Bayesian rules to infer the probability that proteins are found in the same complex, based on their co-occurrence in interactome data. We adapted this idea to estimate the probability that a candidate pair of interacting proteins form a binary bond.

We combined two simple ideas: First, we treated PPIs as probabilistic events occurring in the microenvironment of spliceosomal sub-complexes. We assumed that two proteins are more likely to remain bound if they share additional binding partners that may stabilize the interaction and/or help to position a protein in a specific domain of the spliceosome. Second, we assumed that two spliceosomal proteins highly correlated at the gene-expression level have a better chance to interact than two proteins that are never or rarely co-expressed.

Sources of PPI evidence

Transitivity: This approximation to the clustering coefficient states that a binary interaction between two proteins is more likely if they share a substantial number of interacting partners [3]. This is analogous to the suggest-a-friend application of the social network Facebook. In a customary browsing session, Facebook will suggest new connections (or friends) by predicting people the user may know, based on the expectation that two users are likely to have friends in common.

Thus, considering the modular nature of the spliceosome, we assumed that two proteins are more likely to remain bound if they share a substantial number of binding partners. This may help stabilize the interaction and/or position a protein in a specific domain of the spliceosome. For example, if protein ‘A’ is presumed to bind protein ‘B’, when both ‘A’ and ‘B’ are known to interact with protein ‘C’, the likelihood that ‘A’ and ‘B’ are in physical proximity increases.

In formal terms, given a graph where each vertex v is connected to a group of interacting vertices k, two vertices v_i and v_j show transitivity if there is a number of common vertices k_{ij} within their immediately connected neighbors. In an undirected graph, the transitivity coefficient T_{ij} is given by the number of links shared between the vertices v_i and v_j, divided by the total number of vertices with which they interact.

$$T_{ij} = \frac{2K_{ij}}{K_i + K_j}$$

To calculate transitivity scores, we combined a compendium of 601 binary PPIs from a recent Y2H study focused on the spliceosome [4] with additional annotations from the Human Protein Repository Database (HPRD, http://www.hprd.org/) which contains 37,231 binary PPIs (excluding self-binding) accounting for 9,232 human proteins.

Microarray co-expression: A pair of genes co-expressed at the mRNA level has a better chance to interact at the protein level. Strong co-expression may also indicate that the genes are “committed” by having similar biological functions or being controlled by overlapping regulators [5]. Hence, co-expression profiles can be used to distinguish genuine from spurious PPIs, which are common in interactome data [6].

Pre-mRNA substrates can differ in their requirements for spliceosomal components, as both spliceosomal components and pre-mRNAs can show differential expression across human tissues [7]. Hence, if protein ‘A’ is known to bind protein ‘C’ in tissue ‘x’ but not in tissue ‘y’; and

conversely, protein ‘B’ binds protein ‘C’ in tissue ‘y’ but not in tissue ‘x’, then the interactions A-C and B-C are not good predictors of the interaction A-B.

We quantified the co-expression between interrogated protein pairs using Pearson correlation coefficient (PCC). We used the microarray “Human Atlas” [8] U133A/GNF1H from BioGPS (http://biogps.org/). This dataset contains 85 normal human tissues samples for 13,698 genes, represented by 44,776 probes. We chose this dataset because it has been extensively used for expression analysis (>2000 citations to date).

A previous study indicated a significant correlation between mRNA co-expression and PPIs [9]. Overall, we observed good agreement between the interactomic and transcriptomic datasets selected for our study. ~85% of the PPI pairs in HPRD were also covered in the “Gene Atlas”. In addition, the median PCC of the interacting pairs in HPRD was 0.15. This value was significantly higher than the median PCC computed in five decoy HPRD databases (0.074 to 0.077 Wilcoxon p<8.8E-250) obtained by random shuffling of the edges in HPRD.

Bayesian model

According to the Bayes rule, the posterior probability that a pair of proteins is connected through a binary interaction (P_{in}) can be formulated as:

$$P_{in} = P(e_{ij} = 1|D) = \frac{P(D|e_{ij} = 1) * P(e_{ij} = 1)}{\left(P(D|e_{ij} = 1) * P(e_{ij} = 1)\right) + (P(D|e_{ij} = 0) * P(e_{ij} = 0))}$$

Where $P(e_{ij}=1)$ is the prior probability that a pair of co-immunoprecipitating proteins are directly bound to each other and $P(e_{ij}=0)$ is the complementary value $1-P(e_{ij}=1)$. $P(D| e_{ij}=1)$ is the likelihood that given a series of evidences D, the two proteins are physically bound, whereas $P(D| e_{ij}=0)$ indicates that given D, the two proteins are not bound. Note that $P(D| e_{ij}=1)$ and $P(D| e_{ij}=0)$ are not necessarily complementary; we discuss this idea in greater detail below.

As mentioned above, we chose to use transitivity (*T*) and co-expression (*C*) as evidence to support binary PPIs. Because both metrics are independent, we can assume that the probability of a set of evidences D is given by the product of the probabilities of T and C.

$$P(D) = P(T) * P(C)$$

From here, we deduce:

$$P(D|e = 1) = P(T|e = 1) * P(C|e = 1)$$

$$P(D|e = 0) = P(T|e = 0) * P(C|e = 0)$$

We constructed models of conditional probability for T and C using HPRD to represent true binding instances (*e=1*), (Figure S8A, C) and a "decoy" HPRD (dHPRD) to represent non-binding instances (*e=0*) (Figure S8B, D). The latter was constructed by merging five shuffled versions of HPRD. Consequently, dHPRD is 5 times larger than the original HPRD. This was intended to generate a substantial body of T values above zero, and positive PCCs. We normalized each dataset, derived cumulative distributions for T and C, and finally, assigned conditional probabilities.

These conditional probabilities can be further written as:

$$P(T|e = 1) = P(T \geq t; e = 1)$$

$$P(C|e = 1) = P(C \geq c; e = 1)$$

$$P(T|e = 0) = P(T < t; e = 0)$$

$$P(C|e = 0) = P(C < c; e = 0)$$

In other words, this is the probability that T (or C) is equal or higher than a given value *t* (or *c*) as measured in a true interactome, or the probability that T (or C) is lower than a given value *t* (or *c*), as measured in a decoy interactome.

We defined $P(e=1)$ as the probability that a pair of randomly pooled proteins directly bind to each other.

$$P(e=1)=\frac{2I}{V(V-1)}$$

Where *V* is the total number of proteins in HPRD and *I* is the number of PPIs annotated in HPRD.

In short, $P(e=1)$ corresponds to the number of annotated interactions, divided by the number of potential interactions in HPRD. Conversely, we defined:

$$P(e=0)=1-P(e=1)$$

In the current assembly of HPRD, $P(e=1)$ equals 8×10^{-4}. A previous study reported that the probability that two pooled proteins are members of the same complex is 7×10^{-3} [2]. Therefore a pair of proteins has 10 times greater chance of coexisting in the same complex, than directly binding to each other, further supporting the need for a probabilistic tool to reconstruct PPI networks derived from IPMS.

Supplementary References

1. Liu ZP, Chen L: **Proteome-wide prediction of protein-protein interactions from high-throughput data.** *Protein Cell* 2012, **3:**508-520.
2. Asthana S, King OD, Gibbons FD, Roth FP: **Predicting protein complex membership using probabilistic network reliability.** *Genome Res* 2004, **14:**1170-1175.
3. Wasserman S, and Faust, K: **Social Network Analysis: Methods and Applications.** *Cambridge: Cambridge University Press* 1994.
4. Hegele A, Kamburov A, Grossmann A, Sourlis C, Wowro S, Weimann M, Will CL, Pena V, Luhrmann R, Stelzl U: **Dynamic protein-protein interaction wiring of the human spliceosome.** *Mol Cell* 2012, **45:**567-580.
5. Allocco DJ, Kohane IS, Butte AJ: **Quantifying the relationship between co-expression, co-regulation and gene function.** *BMC Bioinformatics* 2004, **5:**18.

6. von Mering C, Krause R, Snel B, Cornell M, Oliver SG, Fields S, Bork P: **Comparative assessment of large-scale data sets of protein-protein interactions.** *Nature* 2002, **417:**399-403.
7. Will CL, Luhrmann R: **Spliceosome structure and function.** *Cold Spring Harb Perspect Biol* 2011, **3**.
8. Su AI, Wiltshire T, Batalov S, Lapp H, Ching KA, Block D, Zhang J, Soden R, Hayakawa M, Kreiman G, et al: **A gene atlas of the mouse and human protein-encoding transcriptomes.** *Proc Natl Acad Sci U S A* 2004, **101:**6062-6067.
9. Soong TT, Wrzeszczynski KO, Rost B: **Physical protein-protein interactions predicted from microarrays.** *Bioinformatics* 2008, **24:**2608-2614.
