# Supplementary figures and images for: Differential connectivity of splicing activators and repressors to the human spliceosome
Source: Genome Biol. 2015 Jun 6;16(1):119. doi: 10.1186/s13059-015-0682-5 (PMC4502471; doi:10.1186/s13059-015-0682-5)

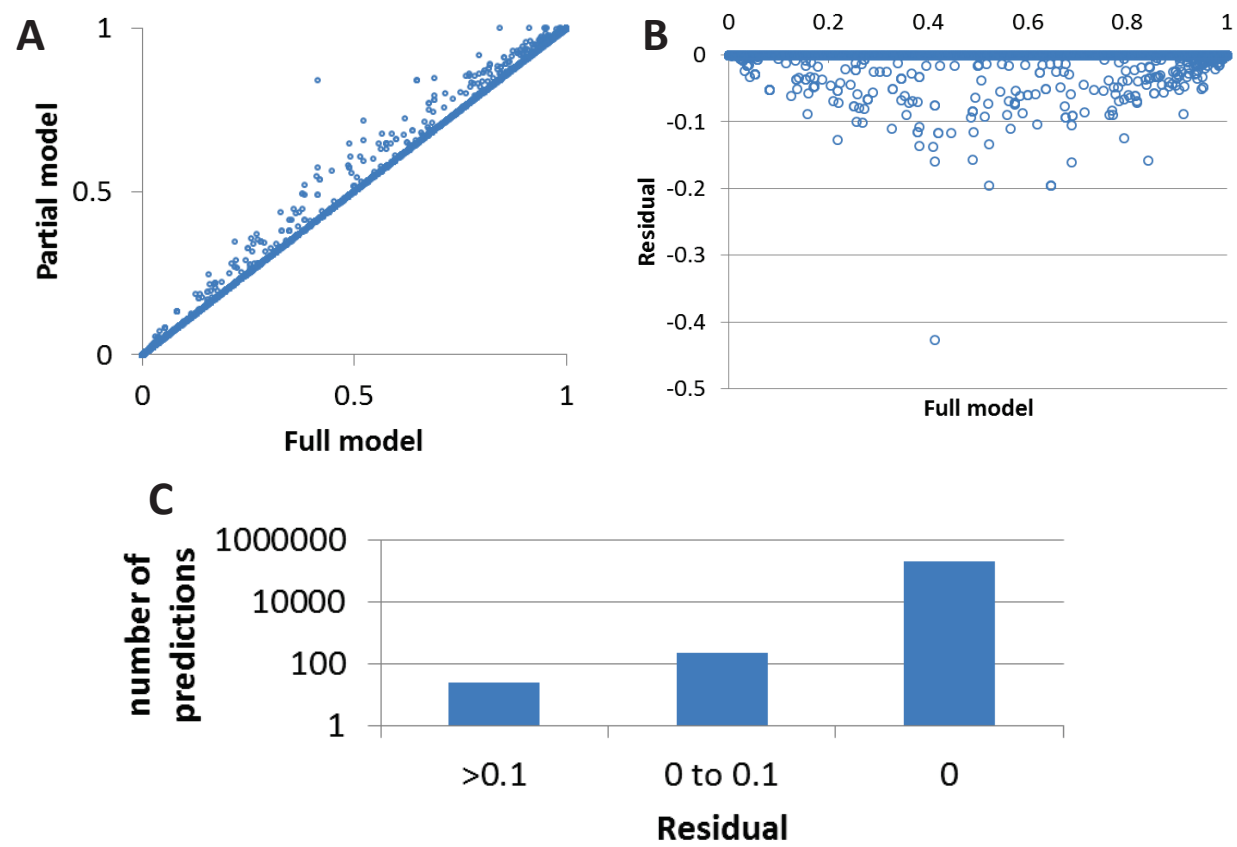

Figure S1

Supplement: Additional file 4: Figure S1. — Effect of direct PPIs on the PS-network. The effect of excluding direct PPI annotations, as opposed to treating them equally to neighboring interactions, for transitivity estimation based on Y2H data. We computed P in scores with direct PPIs (Full model) or without (Partial model). (A) Correlation between P in using vs. ignoring direct PPIs. (B) Residual P in between Full vs. Partial models. (C) Histogram of the distribution of residual scores. [file 13059_2015_682_MOESM4_ESM.pdf]

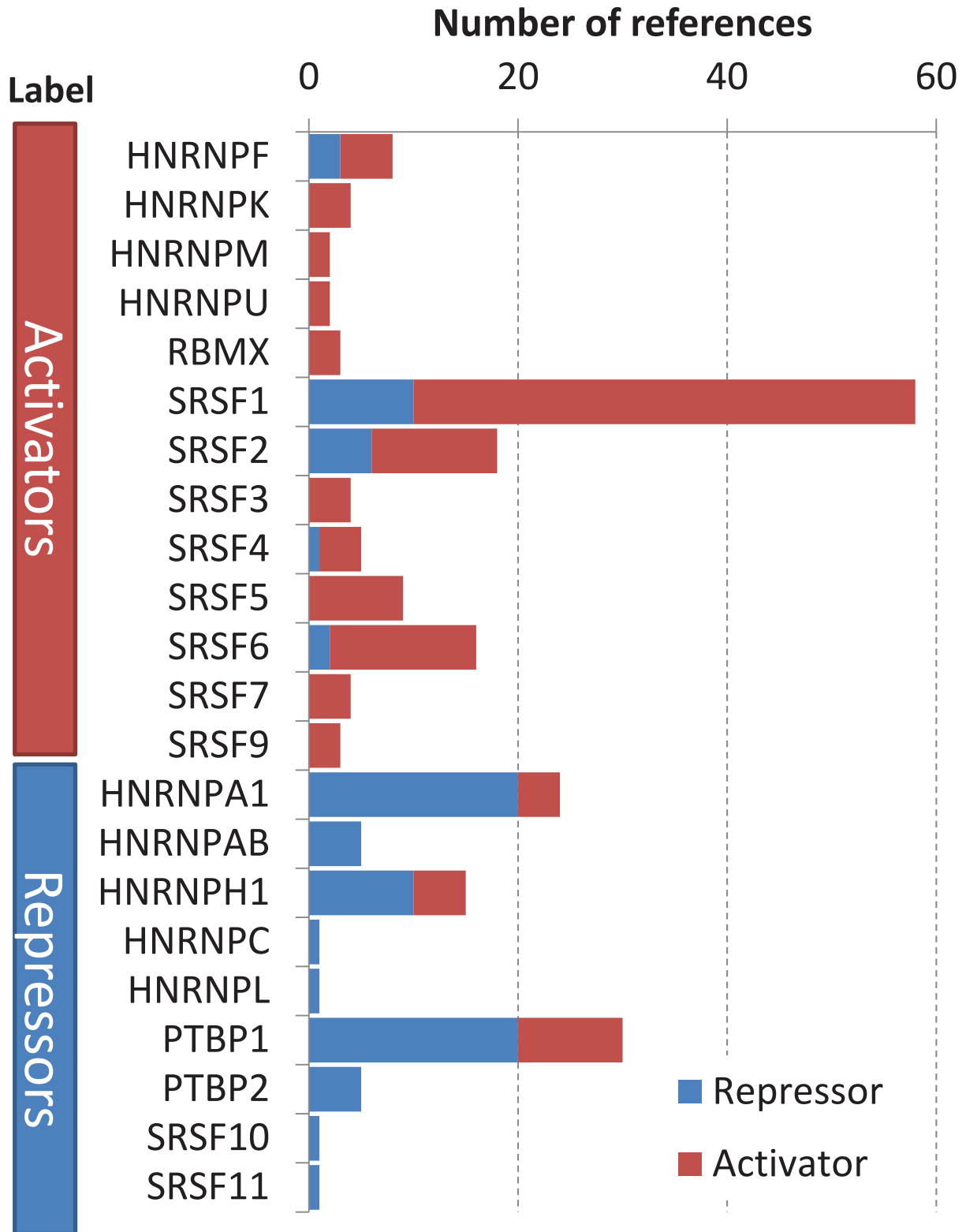

Figure S2

Supplement: Additional file 6: Figure S2. — Classification of SR proteins and hnRNPs into activators and repressors. Based on annotations from RegRNA ([26], Additional file 5: Table S3). The bar plot shows the number of literature references supporting a splicing factor’s activity as activator (red) or repressor (blue). The labels activator or repressors were assigned based on the best supported function of each protein. Labels are shown on the left side of the chart. [file 13059_2015_682_MOESM6_ESM.pdf]

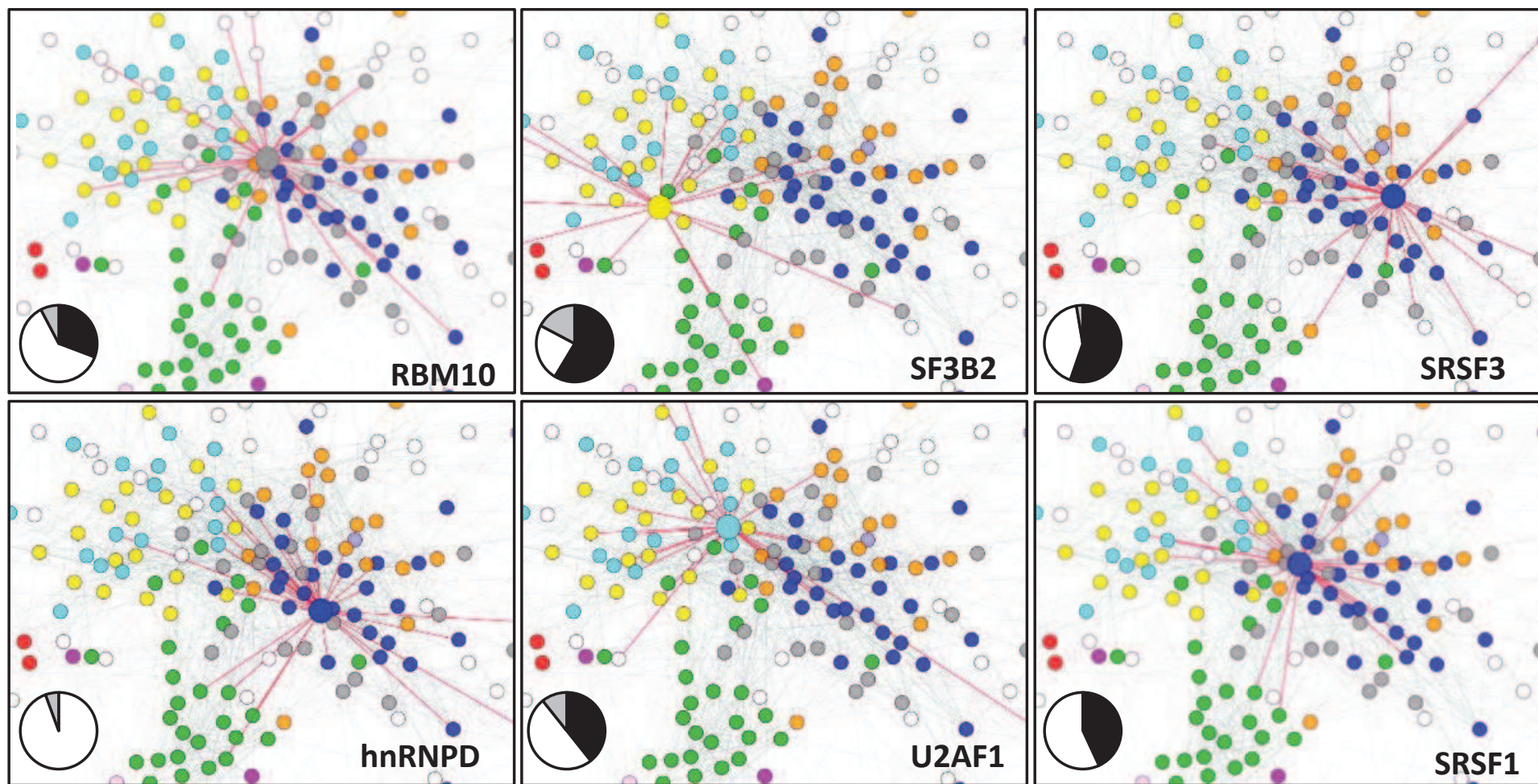

Figure S3

Supplement: Additional file 10: Figure S3. — PPIs formed by top-centrality spliceosomal proteins. See legend from Fig. 4b. [file 13059_2015_682_MOESM10_ESM.pdf]

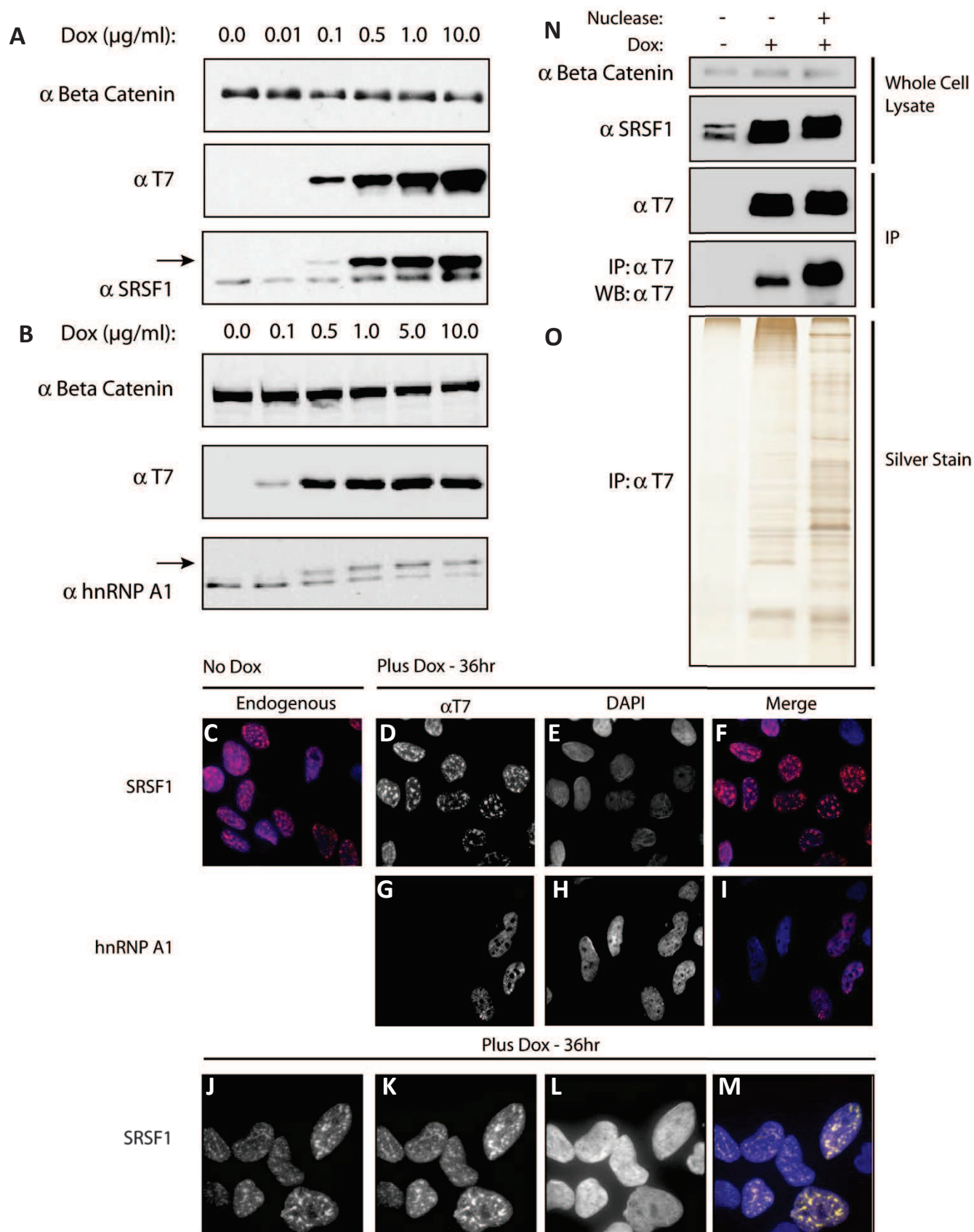

Figure S4

Supplement: Additional file 11: Figure S4. — Immunoprecipitation of SRSF1 and hnRPA1. (A, B) Induced expression of SRSF1 and hnRNPA1 in HeLa cells. (A) HeLa T7-SRSF1 and (B) HeLa T7-hnRNPA1 cells were induced for 36 h with increasing Dox concentrations (from 0.01 to 10.0 μg/mL). Whole-cell lysates were analyzed by western blotting. T7-tagged proteins are marked with an arrow to distinguish them from endogenous proteins. β-catenin was used as a loading control. All cell lines showed a linear response to Dox when probed with endogenous-protein and T7-tag antibodies, although the levels of the T7-tagged splicing factors varied between cell lines. We used 36 h of induction at 0.1 μg/mL Dox for T7-SRSF1, and 0.5 μg/mL Dox for hnRNPA1. (C-M) Cellular localization of induced HeLa TT-SRSF1 and hnRNP A1 is consistent with expression of the endogenous proteins. (C) Indirect immunofluorescence of endogenous (uninduced) SRSF1, (D-F) induced T7-tagged SRSF1 and (G-I) hnRNP A1. (J-M) Co-staining of endogenous SRSF1 (AK-96) and induced T7-SRSF1. Cells were induced with Dox for 36 h at 0.1 μg/mL (SRSF1) and 0.5 μg/mL (hnRNP A1). DNA was stained with DAPI. (N, O) T7-SRSF1 immunoprecipitation (IP) with nuclease treatment: (N) Whole-cell lysates of HeLa TT-SRSF1 cells, with and without nuclease treatment. Cells were induced with Dox for 36 h at 0.1 μg/mL. Nuclease consists of RNases A and T1, plus Benzonase. (Middle) Co-IP of T7-SRSF1 in the presence or absence of nuclease. Co-IP was performed in the presence of 200 mM NaCl. (O) Silver stain of immunoprecipitates. [file 13059_2015_682_MOESM11_ESM.pdf]

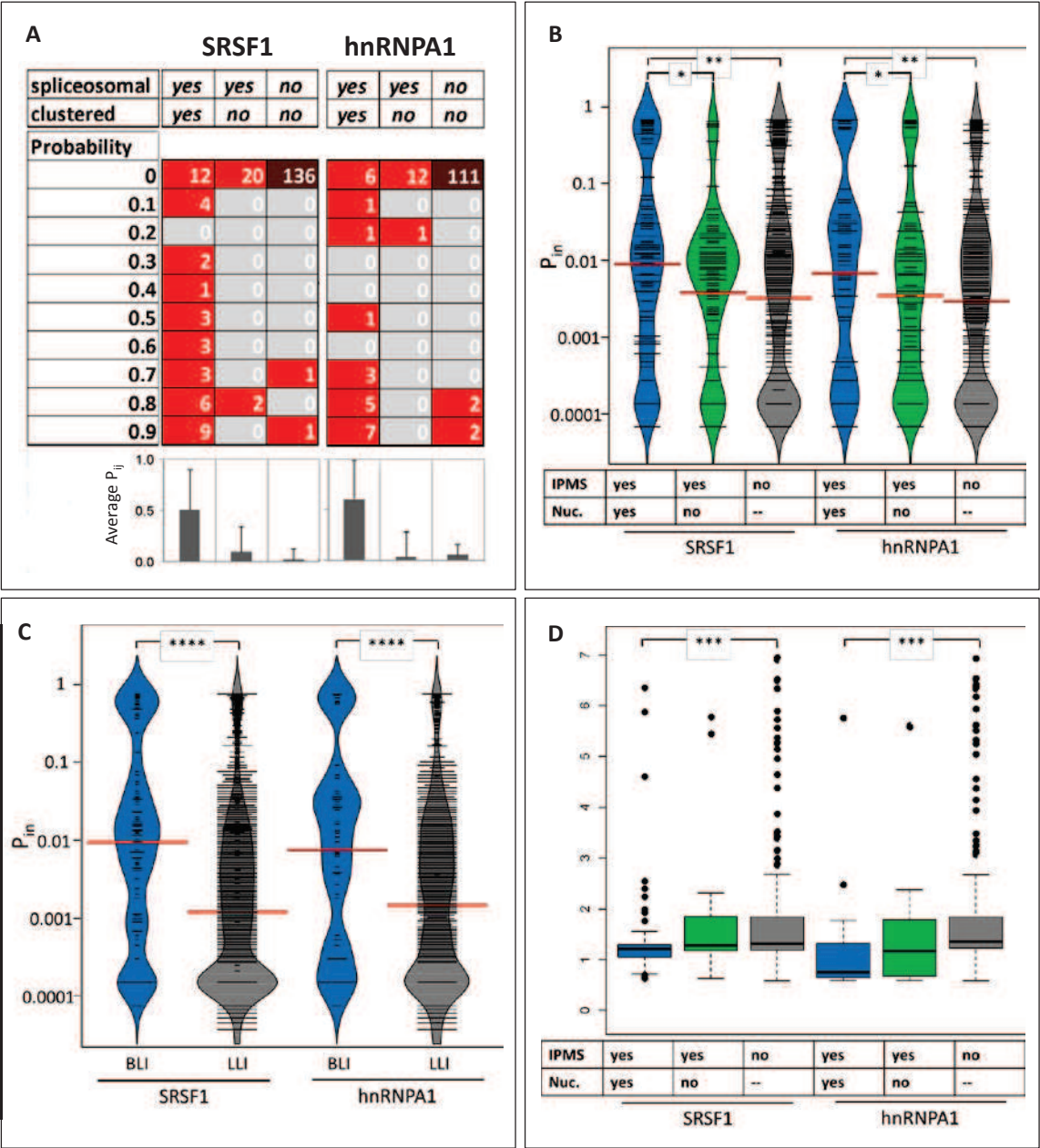

Figure S5

Supplement: Additional file 13: Figure S5. — High-probability PPIs enriched by IP-MS (continued from Fig. 5). (A) The colored tables show the distribution of P in scores for SRSF1 and hnRNPA1 co-purified proteins. These were divided into three categories, depending on whether they are spliceosomal proteins assigned to specific FCs (spliceosomal, clustered) unassigned to FCs (spliceosomal, non-clustered) or non-spliceosomal. The average P in scores for each category are shown at the bottom. (B) Beanplot showing P in distribution of bait-ligand interactions for SRSF1 and hnRNPA1 for IP-MS with (blue) or without (green) nuclease, and for the remaining spliceosomal proteins not identified by IP-MS (gray). Red lines indicate mean values. (C) Similar to B, comparing Pin values from nuclease resistant bait-ligand interactions (blue) to those of ligand-ligand interactions (black). (D) Boxplot showing the distribution of weighted shortest path lengths for either SRSF1 or hnRNPA1 co-purified proteins with (blue) or without (green) nuclease treatment. The remaining spliceosomal proteins that failed to be purified are marked in gray. Wilcoxon test P values are shown in B-D represented by stars as follows: *P <0.05, **P <0.01, ***P <10−3, and ****P <10−5. [file 13059_2015_682_MOESM13_ESM.pdf]

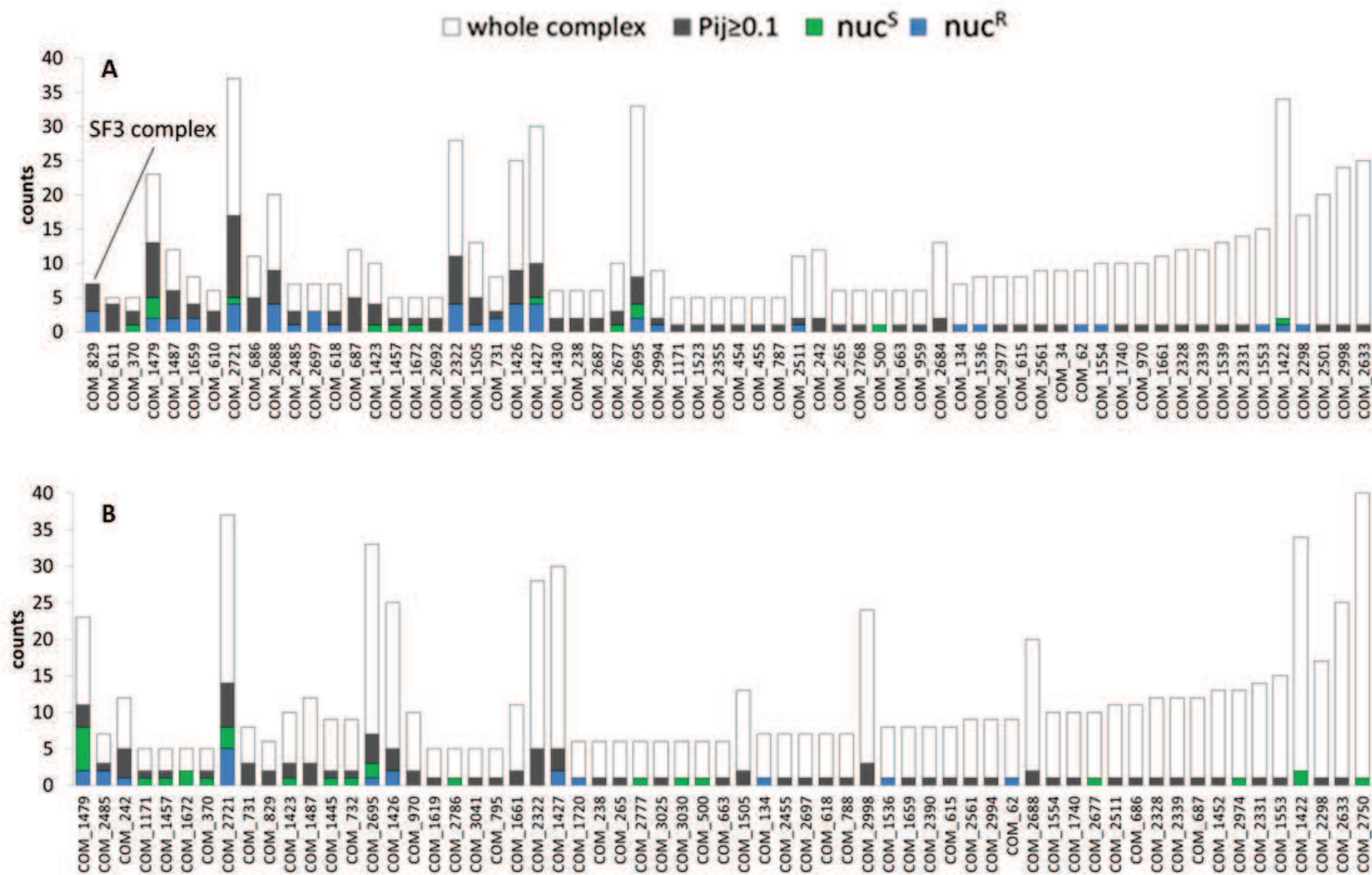

Figure S6

Supplement: Additional file 14: Figure S6. — Complex-composition analysis. Distribution of predicted and co-purified PPIs for (A) SRSF1 and (B) hnRNPA1 among protein complexes annotated in HPRD. [file 13059_2015_682_MOESM14_ESM.pdf]

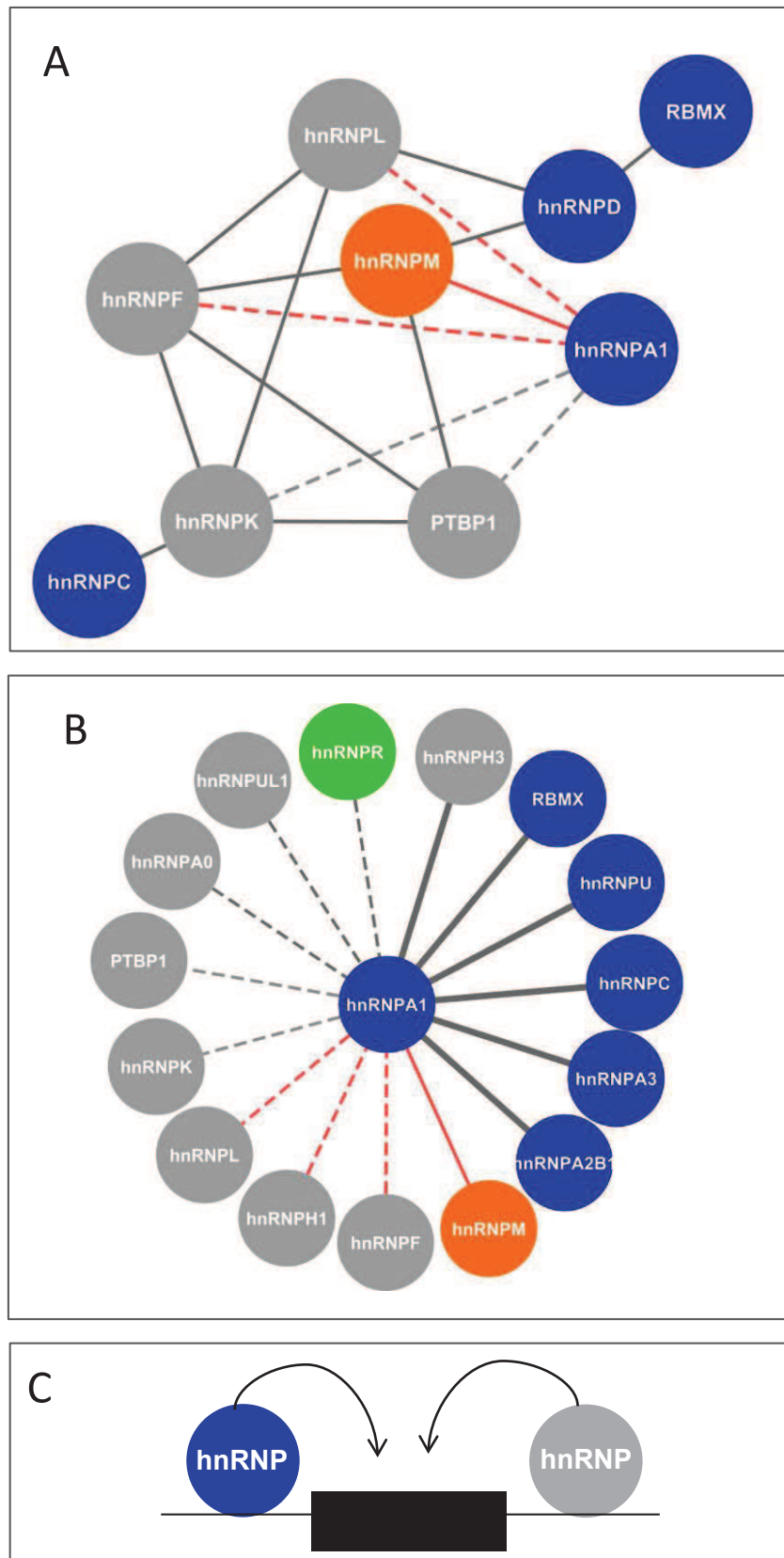

Figure S7

Supplement: Additional file 15: Figure S7. — Physical and regulatory interactions among hnRNPs are mutually exclusive. (A) Regulatory interactions among hnRNPs. The node color indicates the cluster to which each protein belongs, according to the code in Fig. 3d. The edges represent regulatory interactions, as reported by Ke and Chasin [32]. (B) Patterns of interaction between hnRNPA1 and other members of the hnRNP superfamily. Solid edges denote PPIs resistant to nuclease treatment. Dashed edges indicate nuclease-sensitive PPIs. Red edges denote regulatory interactions. The numbers along the edges indicate PI values. (C) Model summarizing co-regulation among hnRNPs. hnRNP pairs from different spliceosomal blocks usually cooperate to regulate alternative splicing. In particular, most proteins belong either to FC5 (blue) or FC9 (gray). [file 13059_2015_682_MOESM15_ESM.pdf]

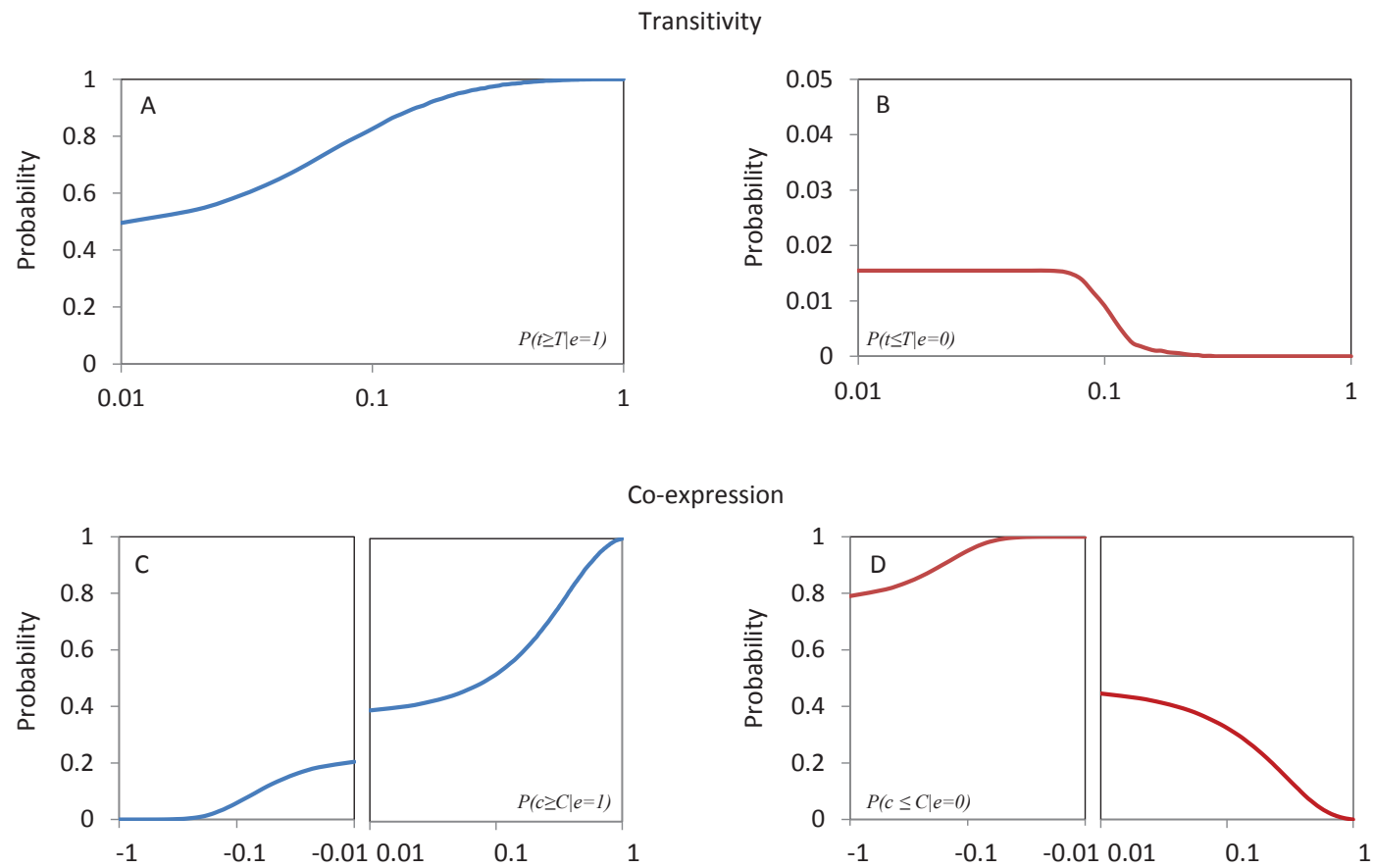

Figure S8

Supplement: Additional file 16: Figure S8. — Conditional probability models. Cumulative distributions for transitivity (A) were calculated using HPRD to represent true binding instances. (B) A similar distribution was derived from a ‘decoy’ HPRD (dHPRD), to represent non-binding instances. In the same way, we generated true (C) and decoy (D) co-expression distributions, by combining both HPRD and dHPRD with the Human U133A/GNF1H microarray dataset. [file 13059_2015_682_MOESM16_ESM.pdf]
