# Supplementary material for: Differential connectivity of splicing activators and repressors to the human spliceosome
Source: Genome Biol. 2015 Jun 6;16(1):119. doi: 10.1186/s13059-015-0682-5 (PMC4502471; doi:10.1186/s13059-015-0682-5)
Supplement: Additional file 7: Table S4. — Topological metrics of the PS-network and deterministic network. [file 13059_2015_682_MOESM7_ESM.pdf]

Table S4: Topological metrics of the PS-network and deterministic network

Topological measures	$P_{(I)}≥0.001$	$P_{(I)}≥0.01$	$P_{(I)}≥0.1$	$P_{(I)}≥0.5$	$P_{(I)}≥0.9$	DET
Diameter	4	5	6	8	10	7
Modularity	0.03	0.05	0.17	0.23	0.49	0.42
Av. shortest path length	1.21	1.88	2.33	2.70	3.74	3.26
Density	0.4	0.24	0.09	0.06	0.02	0.03
Centralization	0.42	0.39	0.28	0.21	0.09	0.09
