# Supplementary material for: Differential connectivity of splicing activators and repressors to the human spliceosome
Source: Genome Biol. 2015 Jun 6;16(1):119. doi: 10.1186/s13059-015-0682-5 (PMC4502471; doi:10.1186/s13059-015-0682-5)
Supplement: Additional file 9: Table S6. — Involvement of high-connectivity spliceosomal proteins in human disease. [file 13059_2015_682_MOESM9_ESM.pdf]

Table S6: Involvement of high-connectivity spliceosomal proteins in human disease

Protein	Description	PMID
SRSF1	Loss of an SRSF1-dependent exonic splicing enhancer leads to downregulation of the SMN1 gene and therefore to Spinal Muscular Atrophy.	16385450
	Splicing factor SRSF1 is upregulated in various human tumors, in part due to amplification of its gene.	17310252
SRSF3	Increased SRSF3 expression in tumor cells is a critical step for tumor initiation, progression, and maintenance.	21179588
	Expression of SRSF3 is associated with malignancy of ovarian tumors but not with stage of invasive epithelial ovarian cancer.	20856201
hnRNPD	hnRNPD proteins are major components of messenger RNA stability complexes which form a novel macromolecular target structure for autoantibodies in rheumatic autoimmune diseases.	18240226
hnRNPF	High levels of hnRNPF are present in premalignant and malignant stages of colorectal cancer, reflecting a role for this protein early in colorectal tumorgenesis.	16424007
hnRNPK	Alternative isoform of hnRNPK found in colonic tumor and surrounding mucosa. It is the first example of an RNA editing event in cancer and its surrounding tissue.	16404425
RBM10	Massively parallel sequencing of X chromosome exons show that TARP syndrome is caused by mutations in the RBM10 gene.	20451169
U2AF1	U2AF1 mutations are frequent in chronic myelomonocytic leukemia and advanced forms of MDS. U2AF1 mutations are predictive for shorter survival.	22323480
	A missense mutation affecting the serine at codon 34 in U2AF1 was recurrently present in 13 out of 150 (8.7%) subjects with de novo MDS. Suggestive evidence of an increased risk of progression to AML.	22158538
	A misspliced form of the cholecystokinin-B/gastrin receptor in pancreatic carcinoma: role of reduced cellular U2AF35 and a suboptimal 3'-splicing site leading to retention of the fourth intron.	11830556
SF3B2	SF3B2 is a biomarker of squamous cell carcinoma.	18973137
