# Supplementary material for: Differential connectivity of splicing activators and repressors to the human spliceosome
Source: Genome Biol. 2015 Jun 6;16(1):119. doi: 10.1186/s13059-015-0682-5 (PMC4502471; doi:10.1186/s13059-015-0682-5)
Supplement: Additional file 17: Table S8 — Summary of MASCOT results. Number of significant peptides detected. [file 13059_2015_682_MOESM17_ESM.pdf]

Table S8: Number of significant peptides detected by MASCOT

SFs	Dox	Nuc	Run1 total	Run2 total	Run1 uniq	Run2 uniq	overlap	%overllap
hnRNPA1	no	no	463	116	390	43	73	16.86
		yes	289	142	201	54	88	34.51
	yes	no	474	250	288	64	186	52.84
		yes	320	209	179	68	141	57.09
SRSF1	no	no	194	458	107	371	87	18.20
		yes	548	415	314	181	234	47.27
	yes	no	276	499	105	328	171	39.49
		yes	609	547	293	231	316	60.31
